# Advanced RSM-Driven Optimisation for Enhancing the Mechanical Performance of FDM-Printed PETG: A Correlated Microstructural and Mechanical Property Investigation

**DOI:** 10.3390/polym17233175

**Published:** 2025-11-29

**Authors:** Rajan Kumaresan, Krishnan Kanny

**Affiliations:** Composite Research Group, Department of Mechanical Engineering, Durban University of Technology, Durban 4001, South Africa

**Keywords:** fused deposition modelling, process parameters, response surface methodology, mechanical properties, microstructural analysis

## Abstract

Fused deposition modelling (FDM) has become a cost-efficient and highly effective technique in 3D printing. Polyethylene terephthalate glycol (PETG) is a prevalent thermoplastic biofilament, and it exhibits resistance to water, heat, and chemicals. It is often regarded as waterproof and possesses exceptional thermal resistance. This study aimed to improve the mechanical properties of PETG by employing a 50% infill density along with certain infill patterns and raster angles for the top, bottom, and interstitial layers. Initially, Response Surface Methodology (RSM) was used to create the regression model by using various parameters; then, it was used to examine the experimental data and find the factors that have a significant impact on mechanical properties. The structural load-carrying behaviour of the specimen was analysed using a Scanning Electron Microscope (SEM). The tensile results showed that the maximum tensile strength attained was 43.09 MPa and the modulus value was 1.18 GPa and the yield strength was 21.01 MPa. The compressive properties showed that the highest strength was 25.90 MPa, and a modulus of 2.87 GPa was attained. The combination of a rectilinear and concentric infill pattern obtained more strength than the other combinations, and the raster angle acted as the most crucial factor on the strength of the specimen. A determination (R^2^) value over 90% signified strong suitability, while the error percentage between estimated and experimental data remained below 5%, showing that the regression values were satisfactory.

## 1. Introduction

The worldwide demand for novel and tailored goods has experienced a notable increase in recent years. To fulfil the demand, the most efficient method for producing functional prototypes of products designed with a computer-aided design (CAD) is through the application of additive manufacturing (AM) techniques [1,2,3]. AM technology, a layered manufacturing approach within the manufacturing sector, facilitates material conservation, the production of customised products, and the creation of intricate shapes in a manner that is comparatively more time-efficient and cost-effective than traditional subtractive manufacturing techniques. In contrast to conventional approaches, it removes the necessity for an additional finishing process like machining [4,5,6,7]. A range of additive manufacturing methods has emerged, each defined by a unique blend of materials and technologies used to incrementally construct layers of the product. This additive manufacturing method is categorised into three main types related to the materials used: liquid, solid, and powder [8]. Material extrusion, sheet lamination, powder bed fusion, vat photopolymerization, material jetting, binder jetting, and directed energy deposition are significant examples of additive manufacturing processes [9,10,11,12,13]. Initially, additive manufacturing found its application in the manufacturing and industrial sectors for the purpose of creating visual representations of products [10]. Advancements in additive manufacturing have enabled the production and use of functional products or individual components as final products [14]. AM has significantly advanced multiple fields, such as aviation, healthcare, food production, and automotive engineering. The automotive, aerospace, and defence sectors have embraced additive manufacturing technology for prototyping and studies in science [15,16,17,18,19]. With the passage of time, additive manufacturing has assumed a vital position in the manufacturing industry, highlighted by its remarkable market growth. In 2023, the assessed value of AM was USD 20.37 billion [20], with an anticipated sustained annual growth rate of 23.3% from 2024 to 2030 [21].

Fused deposition modelling (FDM) or Fused Filament Fabrication (FFF) is a leading 3D printing method in engineering. FDM is prized for its low costs, wide material choice, minimal post-processing, and ability to create complex shapes with internal cavities [22,23]. FDM extrudes molten thermoplastic through a heated nozzle along CAD-derived toolpaths to build parts layer by layer on a heated bed, and its simple, scalable, reliable workflow has driven widespread use in prototyping, education, and industry [24]. Industrial FDM delivers repeatable, engineering-grade prototypes, tooling, and low-volume end-use parts, accelerating iteration and reducing tooling for cost-effective customization across sectors like the automotive and medical sectors [25,26]. It processes a wide range of thermoplastics—PLA, ABS, PETG, nylon, TPU, and fibre-reinforced blends—selected to balance printability, mechanical strength, heat/chemical resistance, and environmental stability [27,28,29]. PETG is a glycol-modified copolyester that exhibits ABS-like strength with PLA-like printability, offering excellent mechanical toughness, chemical and thermal resistance, strong layer adhesion, low warping, transparency, and UV stability for durable engineering parts [30]. PETG balances strength, toughness, and printability, combining PLA’s ease with near-ABS durability, making it well suited to functional parts and housings, with stronger layer adhesion and better chemical and weather/UV resistance than PLA for a longer service life. Because FDM performance is highly process-dependent, optimising PETG parameter settings such as layer height, raster angle, infill, speed, and temperature is crucial to unlock consistent tensile, compressive, and impact strength, including for recycled PETG. PETG’s good mechanical properties make it ideal for prototypes and end-use parts. With a processing temperature of 220–240 °C and density of 1.27–1.28 g/cm^3^, it runs on standard FDM printers while retaining dimensional stability. Previous research shows that FDM’s mechanical properties hinge on process parameters; optimising layer height, print speed, infill density, nozzle temperature, and build orientation significantly improves performance [31,32]. Lower layer heights improve PETG tensile and flexural strength by enhancing interlayer bonding and surface finish. Studies show that the optimal results are around 0.12–0.20 mm, with reduced dimensional error and better structural integrity. Higher infill density increases load-bearing and tensile strength, with major gains up to 40–80% and diminishing returns beyond [33]. Infill pattern also matters; grid, triangular, and cubic patterns outperform honeycomb and gyroid patterns. Nozzle temperature and printing speed have been identified as critical parameters affecting layer adhesion and material flow characteristics. Studies have shown that mid-range temperatures of approximately 235 °C for PETG provide optimal layer bonding [34], while excessive temperatures can lead to material degradation. Similarly, printing speeds around 20–30 mm/s have been found to balance print quality with production efficiency, with higher speeds potentially causing extrusion inconsistencies [35]. In some other studies, FDM parts are shown to be highly anisotropic. Orientation can change mechanical properties by up to 50%, with loading parallel to 0° delivering the highest tensile strength and perpendicular builds lagging due to weak interlayer bonds [31]. Recent studies increasingly use RSM to optimise FDM parameters, consistently pinpointing settings that significantly enhance tensile and compressive strength and improve dimensional accuracy [36]. These previous experimental studies clarify how FDM process parameters govern PETG mechanical performance through comprehensive, standardised testing and RSM-driven designed experiments [37], addressing gaps in parameter–property relationships. FDM generates reproducible baseline tensile, compressive, and impact data across varied conditions [38,39]; uses RSM to optimise layer height, infill density, print speed, and nozzle temperature; develops predictive models capturing main and interaction effects for data-driven parameter selection; establishes performance benchmarks across printing conditions; and identifies parameter settings that enhance properties without compromising manufacturing efficiency or material use [40,41,42], thereby providing actionable guidance that deepens understanding and improves the reliability and performance of FDM-printed PETG for engineering applications while supporting future optimisation.

This study is the continuation of previous studies. Rajan et al. [43] analysed the impact of various parameters such as raster angles and infill patterns (normal parameters (the infill pattern and raster angle remain the same in all the layers)). In this research, the author clearly explained the effect of eight infill patterns and six raster angles; the results suggest that a concentric infill pattern with a 0° raster angle is most suitable for tensile specimens, and a cubic infill pattern with a 45° raster angle is most suitable for compression specimens. In the following work by Rajan et al. [44], the effects of the infill pattern and the raster angle were analysed by varying the top/bottom layers and the intermittent layers (four parameters (changing the inner infill pattern, top/bottom infill pattern, inner raster, and top/bottom raster)). The study clearly explains that by changing the intermittent layers’ infill patterns and raster angles, the maximum strength of 100% infill density can be achieved by using only 50% infill density. From that, in this work, the process parameters utilised were five various factors: an inner layer pattern, an inner layer raster angle, a top layer pattern, a bottom layer pattern, and a top/bottom layer raster angle. Concentric, rectilinear, and line infill patterns were used, and raster angles of 0°, 45°, and 90° were used for this study. The infill density was kept to 50%, and the solid layer count was also kept to 3; the tensile and compressive properties of the PETG specimens were analysed. The design was developed by using the Response Surface Design (RSD) and the RSM technique used for optimisation of the process parameters.

## 2. Materials and Methods

### 2.1. Materials and Machine

PETG thermoplastic polyester was utilised in the FDM technique, and pellets were obtained from PolyLite™ (Polymaker, Suzhou, China) and manufactured by utilising the Filabot EX2 filament extruder (Filabot, Barre, VT, USA). The unprocessed PETG pellets were subjected to melting and extrusion using the extruder. The extrusion procedure was performed at a temperature of 240 °C to guarantee consistent melt flow through the production. The filament was manufactured using a nozzle with a precise diameter of 1.75 ± 0.02 mm, which guaranteed that it was consistently accurate in the dimensions. To ensure filament stability and mitigate the risk of internal defects, the extruded filament underwent cooling in a controlled water bath maintained at room temperature (25 °C). In order to prevent microstructural defects or warping in the filament, the cooling rate was meticulously regulated. Any remaining moisture was then removed by air drying, by maintaining the temperature at 55 to 65 °C for 4–8 h before printing to prevent bubbling, surface defects, and poor layer adhesion. Thermogravimetric analysis was performed in a previous analysis [44], which showed negligible moisture loss (below 100 °C) and a high decomposition onset of 410.5 °C. The specimens were printed utilising the Raise 3D N2 Plus 3D FDM printer (Raise3D Technologies, Stafford, TX, USA) with 0.4 mm nozzle diameters, because of its strong performance qualities and printing engineering-grade, adaptable thermoplastics. Several capabilities of this FDM-based printer are essential for research projects including mechanical performance evaluation of printed components and process improvement of mechanical parts.

### 2.2. Parameters Selection and Optimisation

**Figure 1:**
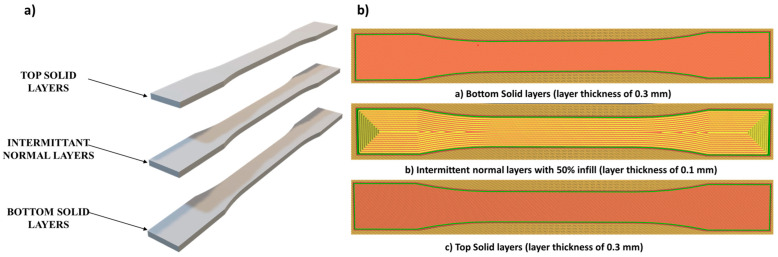
(**a**) Various zones of the specimen. (**b**) Material deposition on various stages of the specimen.

In this research, the specimens were split up into three zones (shown in Figure 1a), top, bottom, and intermittent layers, with the top and the bottom layers being solid layers (0.3 mm) and the intermittent layers being normal (0.1 mm) (shown in Figure 1b). The range of the process parameters used were also divided into two types. To guarantee a reliable process and avoid significant fluctuations that could affect the overall quality of prints, it is crucial to set and uphold uniform printing parameters. Constant parameters for layer thickness were set to 0.1 mm, the initial layer thickness was 0.3 mm, the printing speed was 30 mm/s, the printing temperature was 240 °C, and the print bed temperature was set to 75 °C. Alongside maintaining the values of all the other parameters, the infill density and raster angle were also considered prior to implementing any adjustments. The ranges of printing in the parameters were determined by integrating the maker’s suggestions, findings from studies in the literature, and results from preliminary trial experiments. Based on their significance for mechanical performance, structural anisotropy, and process reliability in FDM printing, three exemplary infill patterns—rectilinear, concentric, and lines—were chosen for this investigation. The patterns establish unique internal architectures that affect stress distribution, inter-layer bonding, and deformation properties under load. Three raster angles (0°, 45°, and 90°) were chosen to assess the influence of layer orientation on the mechanical anisotropy of the printed components. Response Surface Design (RSD) was utilised as the experimental design strategy to optimise printing parameters and reduce the number of experimental trials using Minitab 19 software. RSM is a statistical method that utilises polynomial regression to model the relationships between input variables and output responses, facilitating the identification of optimal process conditions while minimising the number of the physical experiments required. This study employed a Box–Behnken Design to systematically assess the impact of two critical process variables—infill pattern (−1, 0, 1) and raster angle (−1, 0, 1)—on the mechanical performance of FDM-printed specimens. This method facilitates the estimation of main effects, interaction effects, and curvature in the response surface while markedly decreasing the number of specimens needed relative to a full factorial design. The experimental matrix produced by RSM comprised a constrained but statistically significant array of parameter combinations. The developed model using various combinations of the raster angle and infill pattern is shown in Table 1.

### 2.3. Design and Mechanical Testing of FDM-Printed Specimens

The geometric models of the test specimens were developed using PTC Creo Parametric 7.0, in accordance with the ASTM’s applicable standards for testing. The standard for the tensile (ASTM D638) and compressive test (ASTM D695) [44,45] samples were created using Creo7.0 and saved as .STL files. The G-code for machining was generated using the Ideamaker slicing software version 5.2.2. After the preparations of the G-code, the sliced files moved to the FDM printer, and the specimens were printed. After the printing of the specimens, two standard mechanical tests, tensile, and compressive tests were performed to assess the mechanical characteristics of the FDM-printed specimens, following the relevant ASTM standards. All specimens were acclimatised at an ambient temperature (23 ± 2 °C) and relative humidity (50 ± 5%) for a duration of 24 h before testing. The number of specimens printed for each variations totalled *n* = 5. Tensile testing was performed utilising a INSTRON universal testing machine (UTM) (INSTRON, Norwood, MA, USA) with a 50 KN load cell. The specimens underwent testing at a crosshead speed of 10 mm/min, with strain measured via an extensometer affixed at the gauge length. The primary outputs comprised ultimate tensile strength, Young’s modulus, and elongation at break. Compressive testing was conducted using the same machine, and the compressive strength and modulus were evaluated at a speed of 1.3 mm/min. The specimens were meticulously aligned to prevent buckling and to ensure axial loading. Each test was conducted five times per condition to ensure reproducibility, and average values along with standard deviations were reported. Microstructural analysis was carried out by using the Hitachi TM 3030 plus tabletop SEM (Hitachi, Tokyo, Japan) at 5 kV. The fractured PETG samples were mounted on aluminium stubs using carbon tape and sputter-coated with approximately 5 nm of gold (Quorum 150 T coater, Quorum, East Sussex, UK) to ensure charge dissipation and stable high-vacuum imaging.

### 2.4. Design of Experiments (DOEs) and Statistical Analysis

The Design of Experiments (DOEs) approach was systematically utilised to examine the impact of raster angles and infill patterns on the mechanical properties of FDM-printed components. This study examined two factors: infill pattern, categorised into three variables (rectilinear, concentric, and lines), and raster angle, also categorised into three variables (0°, 45°, and 90°). The RSM framework was engaged to minimise the number of experimental trials while effectively acquiring both main effects and interactions. By using the Analysis of Variance (ANOVA), the experimental results were assessed, and the statistical significance of the process parameters was evaluated. A *p*-value below 0.005 was deemed statistically significant, suggesting that the associated factor or interaction exerted a substantial influence on the mechanical response. The R-square (R^2^) values of the developed regression models were calculated to evaluate the goodness of fit. An R^2^ value greater than 90% indicated that the model reliably predicted the experimental outcomes. Pareto charts were employed to visually rank the impacts of each factor and their interactions according to standardised effect size. Regression analysis was performed to create predictive models for tensile and compressive strengths. The models’ accuracy was assessed by comparing predicted values with experimentally observed values, demonstrating significant agreement and confirming the robustness of the developed models.

## 3. Results

In this analysis, except the constant parameters, five parameters were used to print the specimens. In this phase of printing, the parameters for the printing process that varied from the normal parameters were inner pattern, inner raster angle, top layer infill pattern, bottom layer infill pattern, and top/bottom layer raster angle. The outer layer (solid layer) thickness was 0.3 mm, and the inner layer thickness was 0.1 mm. The outer layer count was set as 3 on the top and bottom layers, and the inner normal layer count contained the remaining counts. The outer and inner layer infill patterns varied (concentric, rectilinear, lines) and made the different composition of layers on the specimen. The outer and inner layer raster angles also varied, being 0°, 45°, and 90°. The optimised table was made using the RSM technique, and 46 compositions were made in total. The average tensile and compressive properties of the various compositions of the infill patterns and raster angles from the printed specimen results are shown in Table 2 and Table 3, respectively.

### 3.1. Tensile Properties

#### 3.1.1. Ultimate Tensile Strength

**Figure 2:**
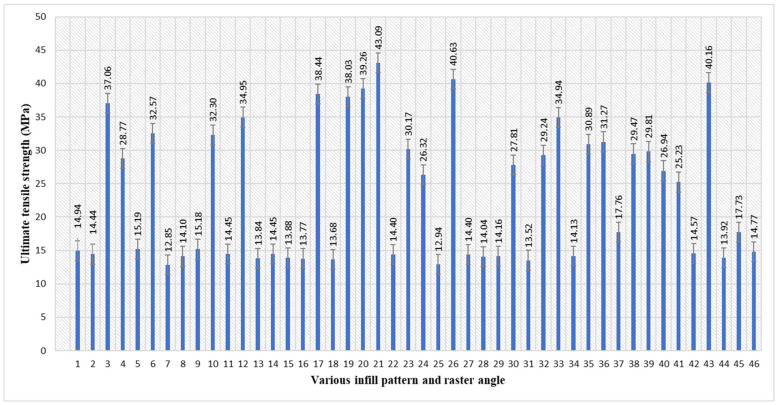
Average ultimate tensile strength of PETG specimens with various infill patterns and raster angles.

The maximum ultimate tensile strength of 43.09 MPa was observed in sample 21, as indicated in Table 2 and Figure 2. The parameters of sample 21 include an inner infill with a rectilinear pattern, an inner raster angle of 45°, a top infill with a rectilinear pattern, and a bottom infill with a concentric pattern, with top and bottom raster angles of 90°. The minimum ultimate tensile strength recorded was 12.85 MPa in sample 7. The parameters of sample 7 include an inner infill with a line pattern, an inner raster angle of 45°, a top infill with a concentric line pattern, and a bottom infill with a line pattern, with top and bottom raster angles of 45°. The rectilinear infill’s highly ordered internal structure, which improves stress distribution along the major loading direction, is responsible for its higher performance when paired with a 0° raster angle [45]. By facilitating effective load transmission between adjoining layers, the alignment of deposited layers minimises premature failure and stress accumulation spots [46]. Conversely, the line-patterned infill demonstrated reduced tensile strength, owing to irregular interlayer adhesion [47], resulting in localised instabilities that promoted fracture initiation and propagation.

#### 3.1.2. Modulus of Elasticity

**Figure 3:**
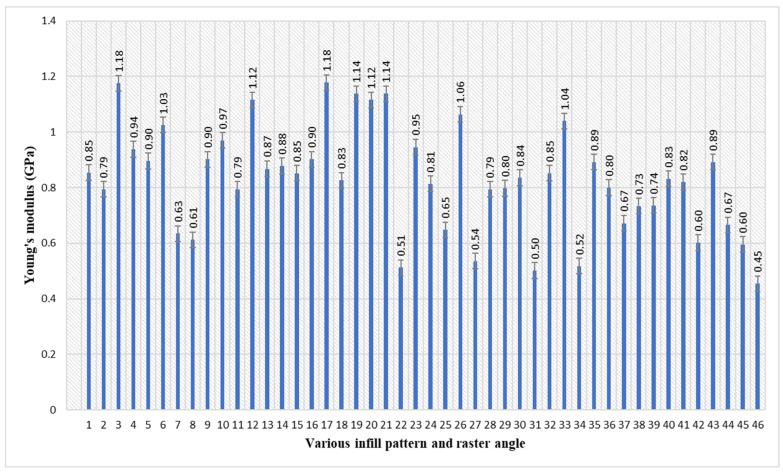
Average elastic modulus of PETG specimens with various infill patterns and raster angles.

As shown in Table 2 and Figure 3, the maximum elastic modulus of 1.18 GPa was attained in samples 3 and 16. The parameters of sample 3 are an inner infill with a rectilinear pattern, with an inner raster angle of 90°, and a top infill with a rectilinear pattern and a bottom infill with a rectilinear pattern, with a top/bottom raster angle of 90°. The parameters of the sample 16 are the inner infill with a rectilinear pattern, with an inner raster angle of 45°, a top infill with a rectilinear pattern, and a bottom infill with a rectilinear pattern, with a top/bottom raster angle of 45°. This guarantees a homogeneous and consistent internal structure across the essential regions of the sample. This structural uniformity reduces internal vulnerabilities [48], resulting in a more consistent and elevated elastic modulus relative to heterogeneous or less structured configurations. The lowest elastic modulus of 0.45 GPa was attained in sample 46. The parameters of sample 7 are an inner infill with a rectilinear pattern, with an inner raster angle of 45°, a top infill with a line pattern, and a bottom infill with a line pattern, with a top/bottom raster angle of 45°. When all layers are set to a single 45° raster angle, the extremely directed line pattern produces a continuous network of weak, diagonal links that run through the sample. The 45° raster angle essentially causes the lowest observable stiffness by focusing the main external load on the part’s weakest structural components, which are the interfaces between the deposited layers of the line pattern.

#### 3.1.3. Yield Strength (0.2% Offset)

**Figure 4:**
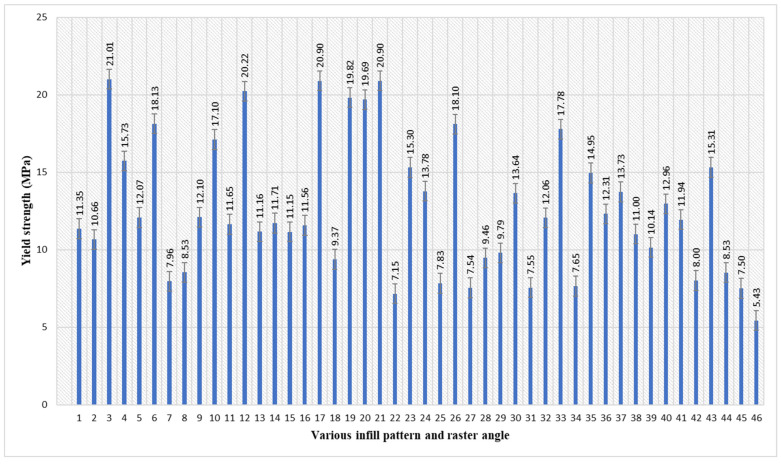
Average yield strength of PETG specimens with various infill patterns and raster angles.

According to Table 2 and Figure 4, the maximum yield strength of 21.01 MPa was attained in sample 3. The parameters of sample 3 are an inner infill with a rectilinear pattern, with an inner raster angle of 90°, a the top infill with a rectilinear pattern, and a bottom infill with a rectilinear pattern, with a top/bottom raster angle of 90°. The layers were aligned as closely as possible, parallel to the primary direction of the applied load. The combination of the rectilinear pattern and the 90° raster angle, which produced the high yield strength. By maximising the cross-sectional area of the material supporting the load, this ideal alignment necessitated the maximum stress needed to cause plastic deformation [49]. The lowest yield strength of 5.43 MPa was attained in sample 46. The parameters of sample 7 are an inner infill with a rectilinear pattern, with an inner raster angle of 45°, a top infill with a line pattern, and a bottom infill with a line pattern, with a top/bottom raster angle of 45°. On the inter bonding layers, the line infill pattern generates a sequence of simple, parallel, disconnected internal lines. This design produces a distinct, predictable, and shear-resistant structure with weak boundaries when it is printed consistently at a single 45° angle [50,51]. Consequently, the 45-degree raster angle, when paired with a directional infill pattern, establishes optimal conditions for failure by low-energy shear/delamination, resulting in the lowest yield strength recorded.

### 3.2. Compressive Properties

#### 3.2.1. Compressive Strength

**Figure 5:**
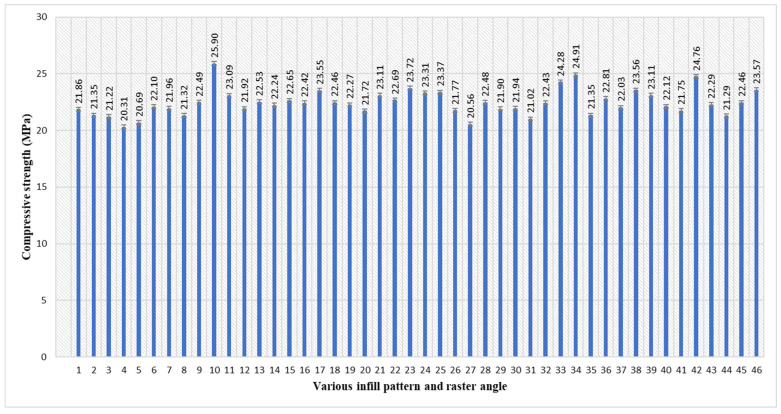
Average compressive strength of PETG specimens with various infill patterns and raster angles (5 parameters).

According to Table 3 and Figure 5, the maximum compressive strength of 25.90 MPa was attained in sample 10. The parameters of sample 10 are an inner infill with a rectilinear pattern, with an inner raster angle of 0°, a top infill with a concentric pattern, and a bottom infill with a rectilinear pattern, with a top/bottom raster angle of 45°. The lowest compressive strength of 20.31 MPa was attained in sample 4. The parameters of sample 4 are an inner infill with a rectilinear pattern, with an inner raster angle of 45°, a top infill with a concentric pattern, and a bottom infill with a line pattern, with a top/bottom raster angle of 45°. Maximum compressive strength arises from the inner rectilinear 0° infill, which forms robust vertical supports that counteract the vertical compressive force [52], while the concentric top layer offers structural confinement to avert premature buckling. The 0° raster angle in the inner infill is typically necessary to create strong, load-bearing columns parallel to the compressive force to achieve maximum strength [53]. By changing this to 45°, the structure is fundamentally weakened against the vertical load.

#### 3.2.2. Compressive Modulus

**Figure 6:**
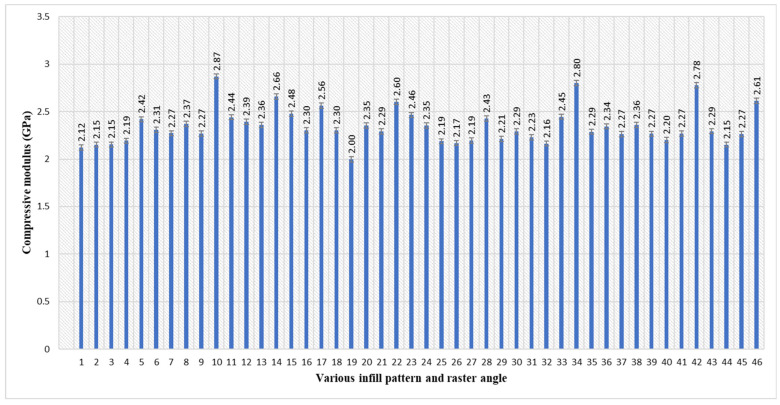
Average compressive modulus of PETG specimens with various infill patterns and raster angles (5 parameters).

The maximum compressive modulus of 2.87 GPa was achieved in sample 10 according to Table 3 and Figure 6. Sample 10 has the parameter combinations of an inner infill with a rectilinear pattern, with an inner raster angle of 0°, a top infill with a concentric pattern, and a bottom infill with a rectilinear pattern, with a top/bottom raster angle of 45°. The minimum compressive modulus of 2.00 GPa was obtained in sample 19. Sample 19 has the parameter combinations of an inner infill with a concentric pattern, with an inner raster angle of 45°, a top infill with a rectilinear pattern, and a bottom infill with a rectilinear pattern, with a top/bottom raster angle of 90°. From this study, the combination of the rectilinear and concentric pattens obtained more strength; this is similar to the results of previous studies [44]. Meanwhile, the lowest compressive modulus was obtained from the same composition. Thus, except for the infill pattern, the raster angle also affected the modulus. In sample 10, the top/bottom raster is 45°, and the inner pattern raster is 0°, but in sample 19, the top and bottom solid-layer raster angle is 90°. Thus, with a lower amount of infill percentage, the 90° raster angle will not observe a more compressive modulus.

### 3.3. Microstructural Behaviour of Various Parameters from Printed PETG Specimens

**Figure 7:**
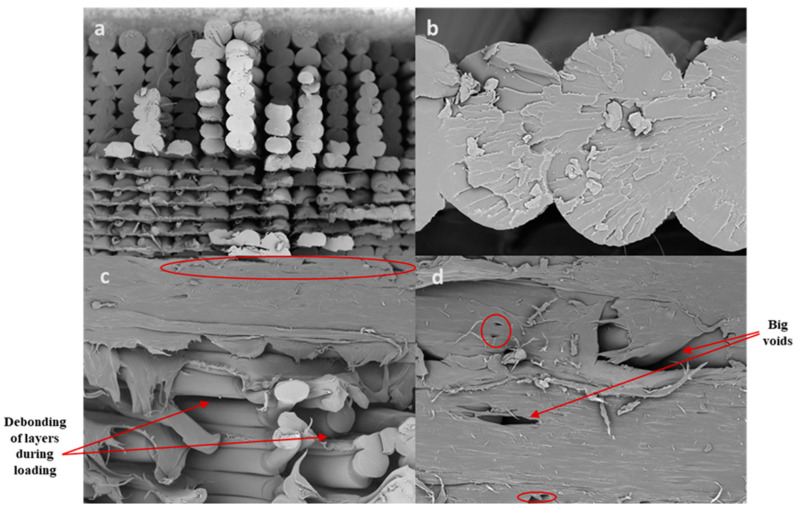
Microstructural characteristics of tensile specimens: (**a**,**b**) specimen 3; (**c**,**d**) specimen 7.

Figure 7 shows the SEM images of PETG specimens, Figure 7a,b show the microscopic images of specimen 3. Figure 7a shows the joining zone of the normal and solid layers; the top of the image shows the solid layers, and the normal layers with a rectilinear infill pattern and a 90° raster angle are shown at the bottom of the figure. From this, during elongation, the linear load (tensile) acted over the layers from end to end so that the load was distributed uniformly. Figure 7b shows the joining zone of the solid layers; it clearly shows that there are zero air gaps, and no voids are formed. Figure 7c,d show the tensile images of specimen 7, which is combination of line and rectilinear infill patterns with a 45° raster angle. Figure 7c shows the joining zone of the rectilinear and line infill patterns. The marked areas clearly show the voids and air gaps in the line infill pattern in Figure 7d, which will clearly affect the tensile strength of the specimen.

**Figure 8:**
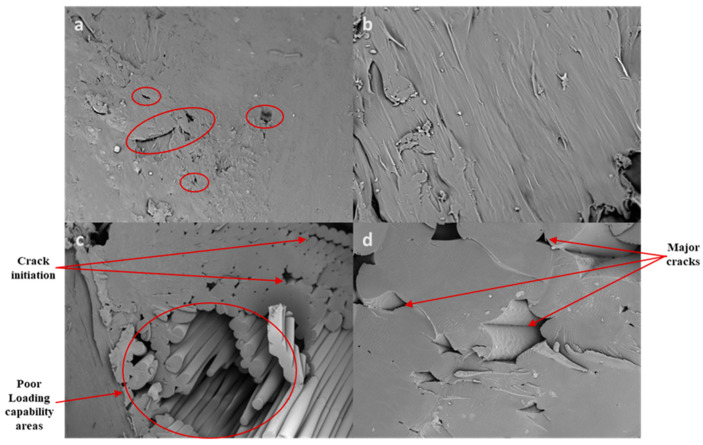
Microstructural characteristics of compressive specimens: (**a**,**b**) specimen 10; (**c**,**d**) specimen 4.

Figure 8 shows the SEM images of specimen 10 (Figure 8a,b) with a combination of rectilinear and concentric patterns and with 0° and 45° angles. Specimen 4 (Figure 8c,d) has a combination of concentric, line, and rectilinear patterns with a 45° raster angle. Figure 8a shows minor cracks after the compression load applied over the specimen, which is not significantly affect the strength. Figure 8b shows a clear surface, which means proper bonding of the layers (zero airgap, without voids) in the specimen and helps obtain a more compressive load. Figure 8c shows the joining zone of the outer and inner patterns (concentric and lines); this clearly shows that improper bonding on the layers and more voids affect the properties. Also, it shows crack initiation between the layers during compressive load, which tests the strength-carrying capability of the specimens. The major cracks in the inner surface of the specimen and air gaps between the layers and voids in the layer shown in Figure 8d, which will lead to more cracks in the inner structure, also affect the mechanical ability of the specimens.

### 3.4. Statistical Analysis

In this chapter, the complexity of FDM specimens is heightened by considering five distinct parameters during the printing process. Statistical analysis becomes even more critical as we navigate the intricate interplay of these additional factors. Through rigorous statistical examination, we aim to unveil the multifaceted relationships between parameters and their collective impact on the properties of FDM specimens. The insights gained from this analysis will inform a nuanced understanding of how to fine-tune FDM processes for advanced applications and improved outcomes. The intricate relationships between tensile strength and two crucial process factors in fused deposition modelling, raster angle and infill pattern, are examined in this chapter. Analysing these two factors statistically helps to clarify the complex dynamics affecting tensile strength and enhance FDM processes for better material performance.

#### 3.4.1. Statistical Analysis on the Tensile Strength

##### Statistical Analysis of UTS

**Figure 9:**
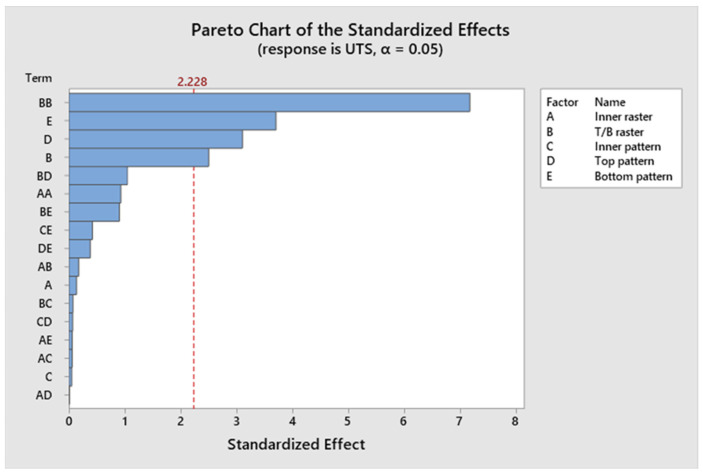
Pareto chart of ultimate tensile strength.

**Figure 10:**
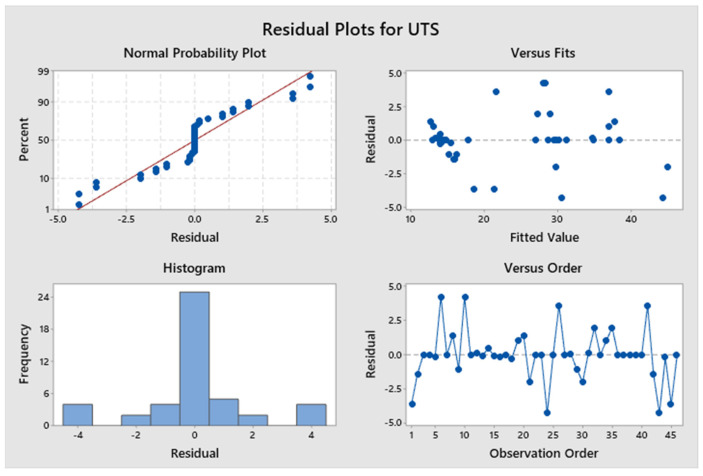
Residual plots for ultimate tensile strength.

**Figure 11:**
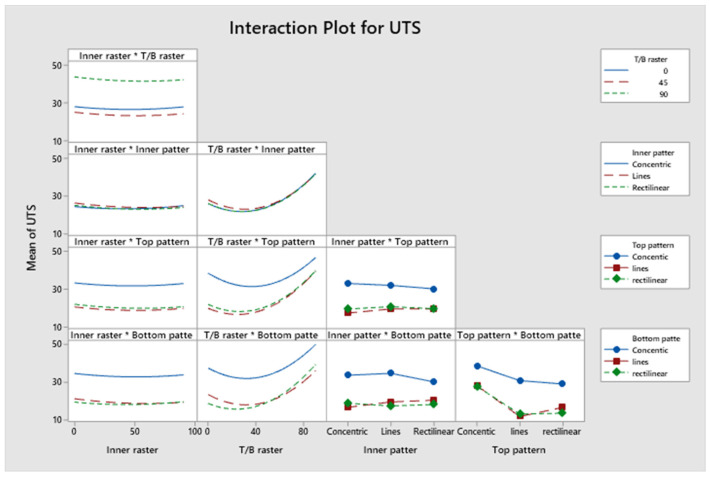
Interaction plots for ultimate tensile strength.

The model, linear interaction effect, top/bottom raster angle, and outer patterns all have *p*-values < 0.05, which is indicative of a statistically significant effect, according to Supplementary Table S1. The *p*-value for Factor A (inner raster angle) is 0.894, and for Factor C (inner pattern), it is 0.961, indicating a lack of statistical significance. Factor D (top infill pattern) constitutes roughly 14.96%, Factor E (bottom pattern) constitutes 16.12%, and aggregate Factor B (top/bottom raster) constitutes 35.76%. Factors C and A contribute 3.17% and 0.5%, respectively. Factors B, D, and E exert a more significant influence than Factors A and C. Figure 9 presents a Pareto chart illustrating the different confidence level parameters. Figure 10 represents the residual plots for the UTS. This diagram validates the statistical assumptions of the UTS regression model. The Normal Probability Plot and Histogram show that the residuals are generally normally distributed and centred near zero, satisfying the normality assumption. The Versus Fits plot demonstrates a random, uniform scatter of residuals around the zero-horizontal line, confirming constant variance (homoscedasticity) and adequate linearity. Finally, the Versus Order plot shows no discernible pattern over the observation sequence, confirming the crucial assumption of independent errors. These discoveries confirm the model’s statistical integrity and reliability. Figure 11 shows the interaction effect of the various parameters in the UTS. Factors E, D, and B significantly contribute to the ultimate tensile strength of the specimen. Also, this indicates that the R^2^ value is 96.63%. A higher R^2^ value indicates a superior fit of the model to the data. The relationship’s significance is indicated by the adjusted R^2^, which is 89.69%. The future mathematical model demonstrates a stronger relationship between the aspect and the response, as indicated by the higher adjusted R^2^ value. The predicted R^2^ indicates that the model achieves an accuracy of 84.85%. Supplementary Table S2 presents the regression equation of UTS in relation to various operating parameters.

Comparison of Experimental and Predicted Ultimate Tensile Strength

**Figure 12:**
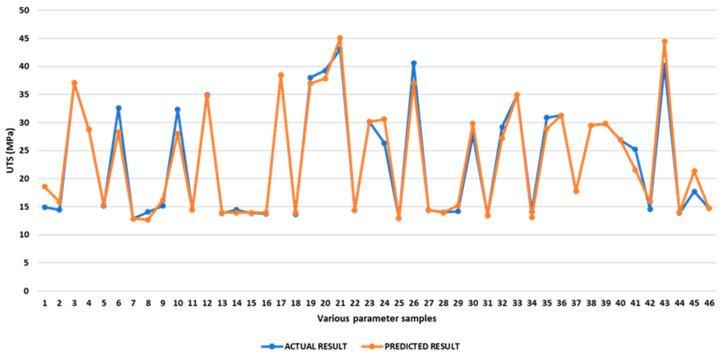
Comparison of experimental and predicted UTS.

The predicted and experimental tensile strength are shown in Supplementary Table S3. Average discrepancies between experimental and predicted UTS are found to be 4.70%, with a range from 0.00% to 24.26%. The optimal UTS was also determined through maximising reactions. The optimal value is determined within the given constraints and range to maximise UTS. As calculated by the optimisation, a UTS of 45.07 MPa is achievable with an inner infill demonstrating a rectilinear pattern, with a raster angle of 45°, a top infill with a rectilinear pattern, and a bottom infill with a concentric pattern and with a 90° raster angle. Figure 12 presents a picture comparing the predicted UTS value with the measured value for various operating parameters.

##### Statistical Analysis of Modulus of Elasticity

**Figure 13:**
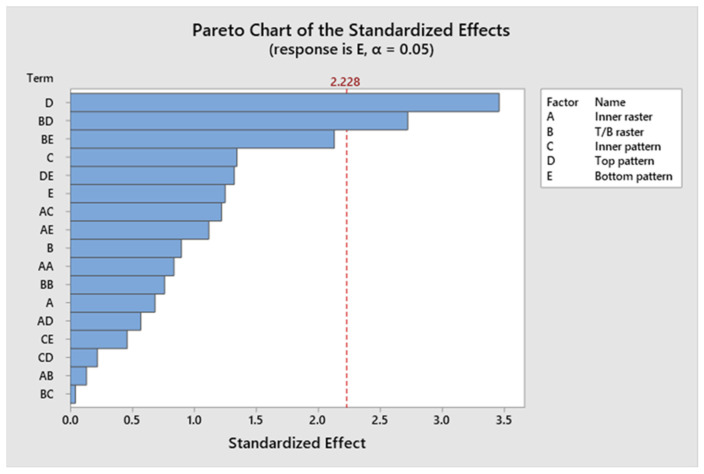
Pareto chart of elastic modulus.

**Figure 14:**
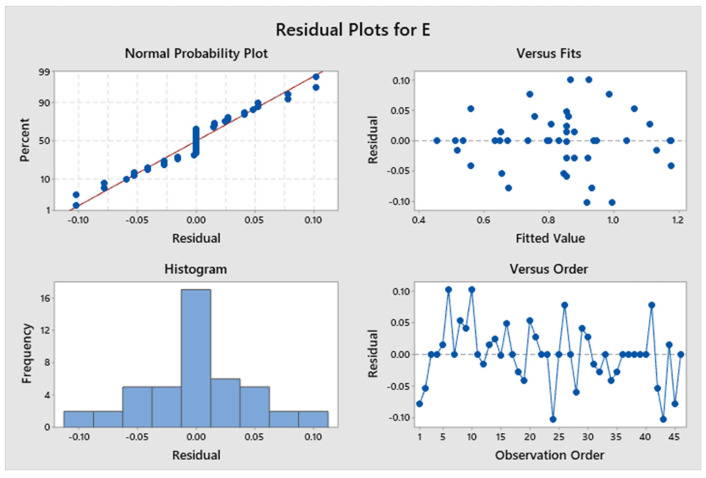
Residual plots for elastic modulus.

**Figure 15:**
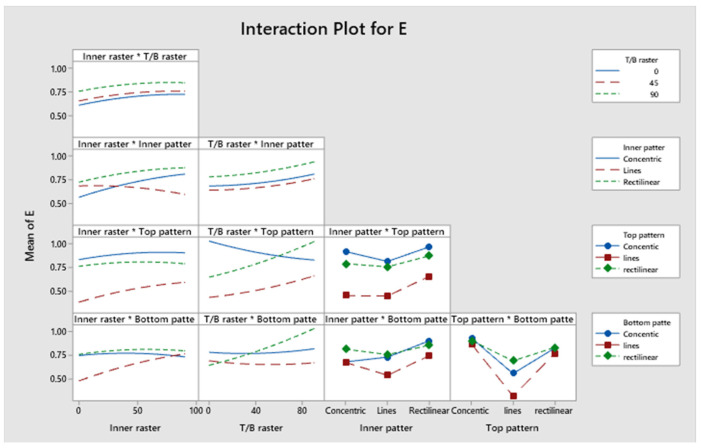
Interaction plot for elastic modulus.

The confidence levels for each parameter are illustrated in a Pareto chart in Figure 13. The elastic modulus of the specimen is predominantly influenced by Factor D. Figure 14 shows the residual plots for E. The Normal Probability Plot and the Histogram show that the residuals are distributed normally and centred around zero, which shows normality. The Versus Fit plot shows random and even data points around the zero-horizontal line, which shows that the relation between the linear and the variance is constant. It is supported by the Versus Order plot, which shows that there is no systematic pattern or time-dependent trend across the 46 experimental runs. This means that the mistakes are independent. The developed model fulfils its basic statistical assumptions. Figure 15 shows the interaction effect of the various parameters on E. Supplementary Table S4 demonstrates that the *p*-values for the model, linear interaction, and the predominant infill patterns are all below 0.05, signifying their substantial influence. However, Factor A exhibits a non-significant *p*-value of 0.510, Factor B has a *p*-value of 0.391, Factor C shows a *p*-value of 0.209, and Factor E presents a *p*-value of 0.240. Approximately 13.86 percent originates from Factor D, 2.94 percent from Factor E, and 51.29 percent is derived from Factor B. Factor A and Factor C contribute 0.50% and 3.17%, respectively. Information is more readily obtained from Factor C and Factor A on the exterior of the item than from the inside. The R^2^ value of 94.14% was indicated; a greater R^2^ value signifies a more accurate alignment between the data and the model. The importance of the correlation is further evidenced by the adjusted R^2^, which stands at 87.72%. The elevated adjusted R^2^ indicates that the proposed mathematical model offers an enhanced understanding of the relationship between the variable and the response. The model’s predicted R^2^ demonstrates an accuracy of 82.16%. The regression equation for the elastic modulus corresponding to various infill patterns is presented in Supplementary Table S5.

Comparison of Experimental and Predicted Young’s Modulus

**Figure 16:**
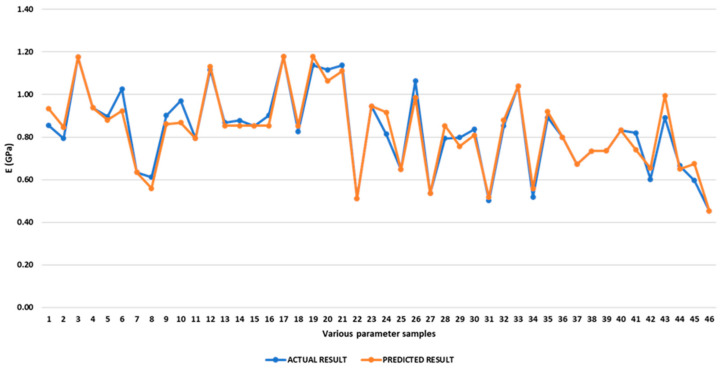
Comparison of experimental and predicted elastic modulus.

Both the theoretical and experimental values for the elastic modulus are presented in Supplementary Table S6. The results show that the deviations between the anticipated and experimental modulus range from 0% to 13.18% and average 3.82%. It was found that maximising responses also gave us the best modulus value. During optimisation, we found that the inner infill with a rectilinear pattern, with a raster angle of 90°, the top infill with a rectilinear pattern, and the bottom infill with a rectilinear pattern and with a raster angle of 90° would result in an elastic modulus of 1.18 GPa. Figure 16 depicts a comparison between the expected and actual elastic modulus for different values of the operating parameters.

##### Statistical Analysis of Yield Strength

**Figure 17:**
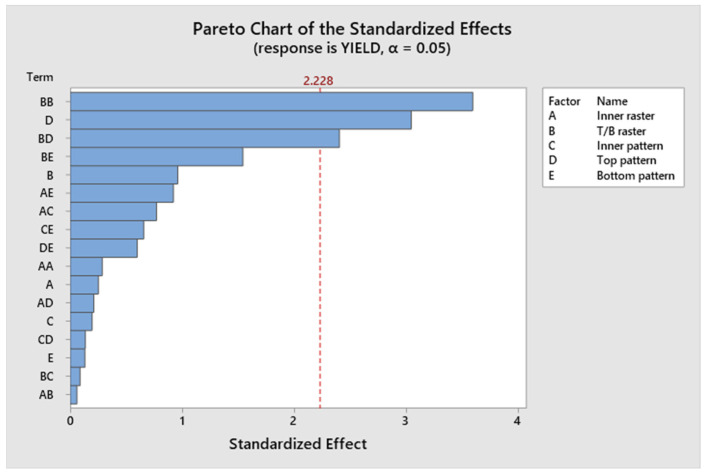
Pareto chart of yield strength.

**Figure 18:**
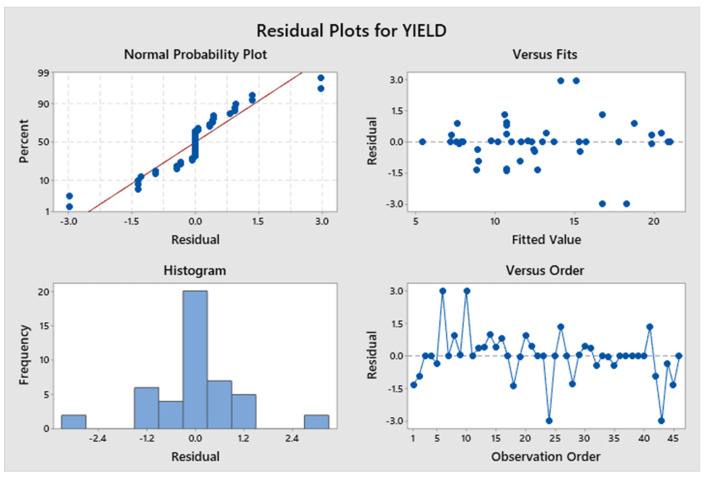
Residual plots for yield strength.

**Figure 19:**
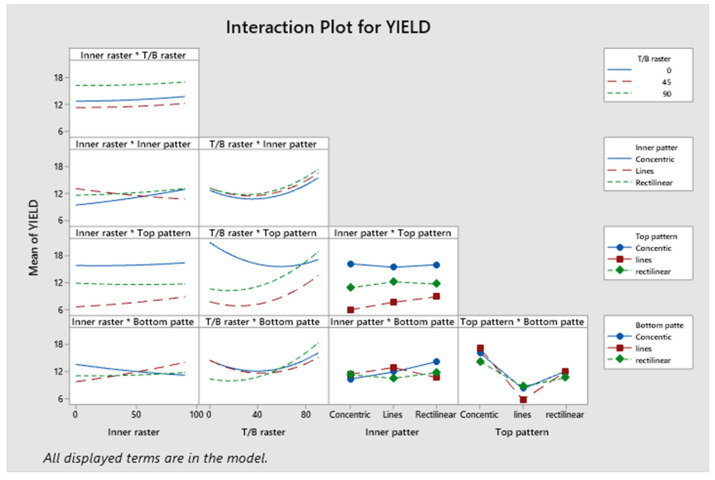
Interaction plot for yield strength.

Figure 17 represents a Pareto chart illustrating the different parameters related to the confidence level. Factor D significantly contributes to the yield strength of the specimen. Figure 18 represents the residual plots for the yield strength. This diagram validates the statistical assumptions of the yield strength regression model. The Normal Probability Plot and Histogram show that the residuals are generally normally distributed and centred near zero, satisfying the normality assumption. The Versus Fits plot demonstrates random, uniform scatter of residuals around the zero-horizontal line, confirming constant variance and adequate linearity. Finally, the Versus Order plot shows no discernible pattern over the observation sequence, confirming the crucial assumption of independent errors. These discoveries confirm the model’s statistical integrity and reliability. Figure 19 shows the interaction plot of the various parameters on the yield strength. The model, linear interaction effect, and Factor D all have *p*-values < 0.05 (0.012), which is indicative of a statistically significant effect, according to Supplementary Table S7. The *p*-value for Factor A is 0.807, for Factor B is 0.361, for Factor C is 0.849, and for Factor E is 0.897, which indicates a lack of statistical significance. Factor D constitutes roughly 16.39%; Factor E, 2.42%; and aggregate Factor B, 45.34%. Factors C and A contribute 1.90% and 0.11%, respectively. Factors B, D, and E exert a more significant influence than Factors A and C. Factor D significantly contributes to the yield strength of the specimen. The value of R^2^ is 94.14%, and a higher R^2^ value indicates a superior fit of the model to the data. The importance of the relationship is demonstrated by the adjusted R^2^, which stands at 87.72%. The proposed mathematical model provides greater insight into the relationship between the aspect and the response, as indicated by the higher adjusted R^2^ value. The predicted R^2^ indicates that the model achieves an accuracy of 82.16% in its predictions. Supplementary Table S8 presents the regression equation correlating yield strength with different operating parameters.

Comparison of Experimental and Predicted Yield Strength

**Figure 20:**
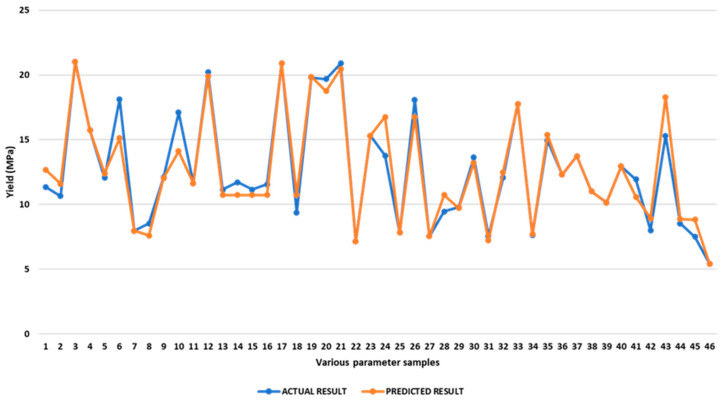
Comparison of experimental and predicted yield strength.

The predicted and experimental yield strength values are shown in Supplementary Table S9. Average discrepancies between experimental and predicted yield strength were found to be 5.16%, with a range from 0.00% to 19.52%. The optimal yield strength was also determined through maximising reactions. The optimal value was determined within the given constraints and range to maximise yield strength. As calculated by optimisation using ANOVA, a yield strength of 20.90 MPa is achievable with an inner infill demonstrating a rectilinear pattern, with a raster angle of 90°, a top infill with a rectilinear pattern, and a bottom infill with a rectilinear pattern, with a 90° raster angle. Figure 20 presents a picture comparing the predicted yield strength value with the measured value for various operating parameters.

#### 3.4.2. Statistical Analysis on the Compressive Properties

##### Statistical Analysis of Compressive Strength

**Figure 21:**
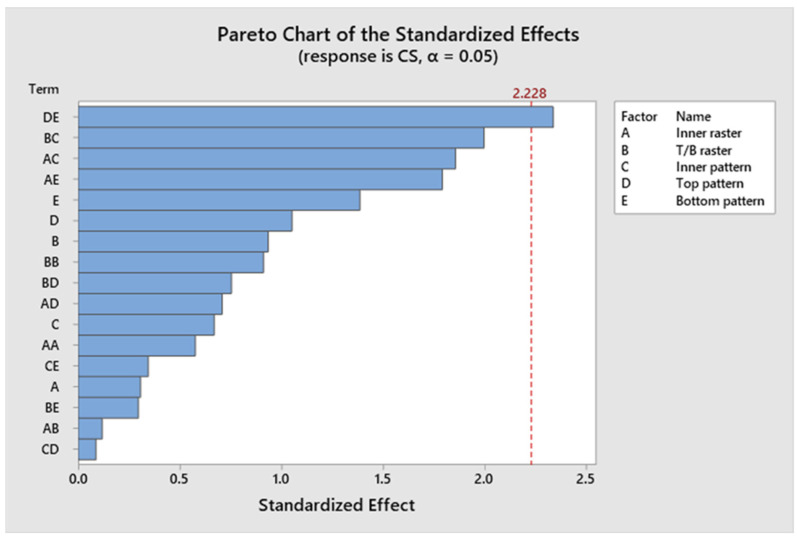
Pareto chart of compressive strength.

**Figure 22:**
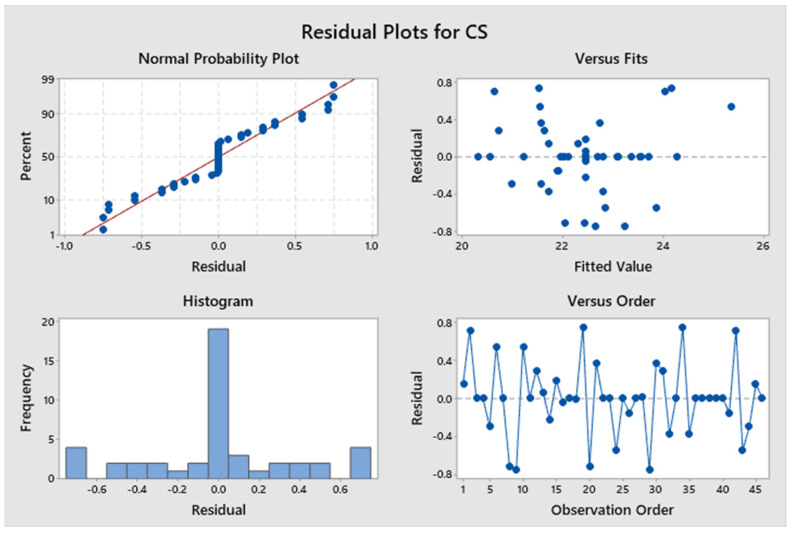
Residual plots for compressive strength.

**Figure 23:**
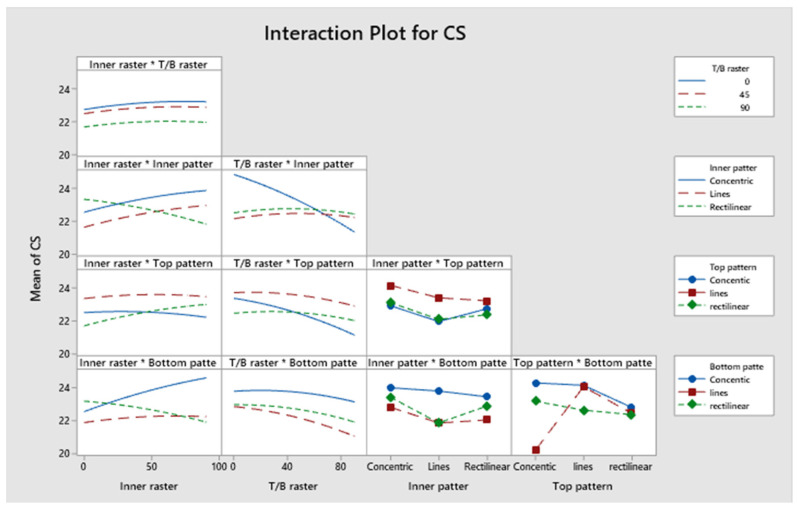
Interaction plot for compressive strength.

Figure 21 illustrates a Pareto chart with different confidence level parameters. None of the components significantly add to the yield strength of the specimen. Figure 22 illustrates residual plots of compressive strength. The Normal Probability Plot and the Histogram both show that the residuals are mostly around zero and follow the straight line closely. This supports the idea that things are normal. The Versus Fits plot shows that the data points are spread out randomly across the range of fitted values. This shows that the data is linear and that the variance is constant (homoscedasticity). Lastly, the Versus Order plot does not show any patterns that happen over time. This shows that mistakes happened at random times during the experiment, which is an important point. These diagnostic checks ensure that the CS prediction model is accurate. According to Supplementary Table S10, not all *p*-values for the model, linear interaction effect, Factor A, Factor B, Factor C, Factor D, and Factor E are below 0.05, indicating a lack of significance. Factor D accounts for approximately 7.46%, Factor E for 3.83%, and Factor B for 0.01%. Factor C and Factor A account for 13.07% and 10.71%, respectively. Factor C and Factor A have a greater impact than Factor B, Factor D, and Factor E. Consequently, Factor C and Factor A influence the specimen’s strength under compression stresses. This also indicates that the R^2^ value is 88.77%. A higher R^2^ indicates a superior fit of the model to the data. The importance of the association is demonstrated by the corrected R^2^, which is 84.46%. The proposed mathematical model demonstrates a stronger association between the variable and the response, as indicated by the elevated adjusted R^2^ value. The projected R^2^ indicates that the model achieves an accuracy of 80.16% in its predictions. Figure 23 shows the interaction effect of the compression strength with various process parameters. Table S11 presents the regression equation correlating compressive strength with various operational parameters.

Comparison of Experimental and Predicted Compressive Strength

**Figure 24:**
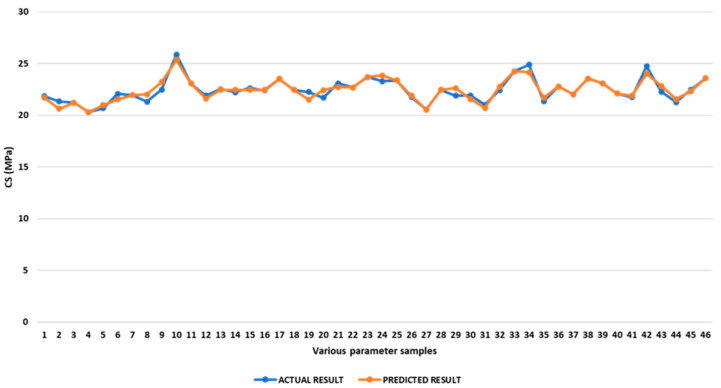
Comparison of experimental and predicted compressive strength.

The predicted and experimental compressive strength values are shown in Supplementary Table S12. Average discrepancies between experimental and predicted compressive strength are found to be 1.15%, with a range from 0.00% to 3.42%. The optimal compressive strength was also determined through maximising reactions. The optimal value is determined within the given constraints and range to maximise compressive strength. As calculated by optimisation, a compressive strength of 25.90 MPa is achievable with an inner infill demonstrating a rectilinear pattern, with a raster angle of 0°, a top infill with a concentric pattern, and a bottom infill with a rectilinear pattern and with a 45° raster angle. Figure 24 presents a picture comparing the predicted compressive strength value with the measured value for various operating parameters.

##### Statistical Analysis of Compressive Modulus

**Figure 25:**
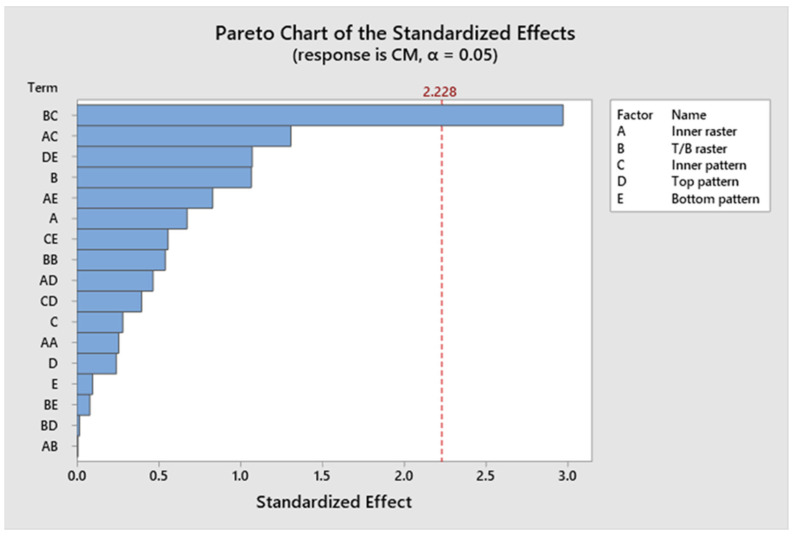
Pareto chart of compressive modulus.

**Figure 26:**
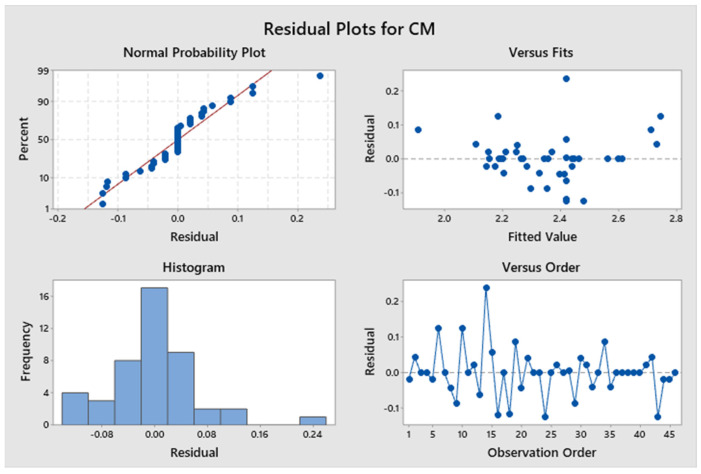
Residual plots for compressive modulus.

**Figure 27:**
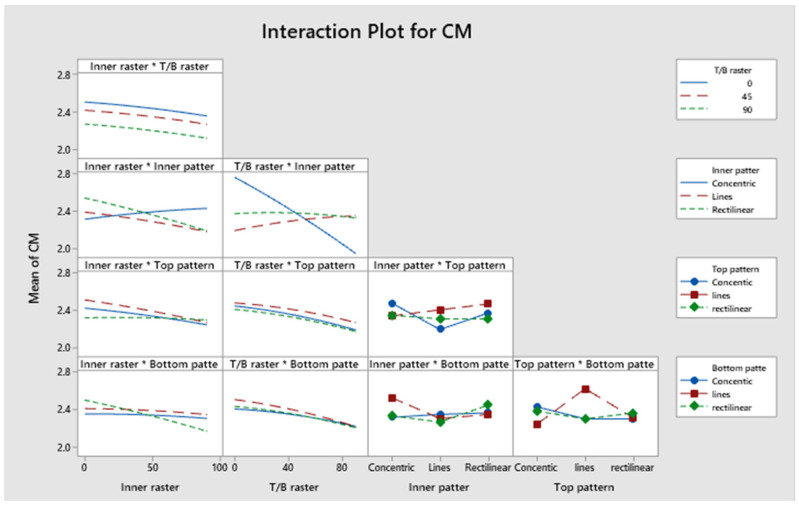
Interaction plot for compressive modulus.

The confidence levels for the separate parameters are illustrated in a Pareto chart in Figure 25. The compressive modulus of the specimen is unaffected by any influences. Figure 26’s residual plots for compressive strength show that the regression model for compressive strength is statistically sound. The Normal Probability Plot and the Histogram both show that the residuals are mostly around zero and follow the straight line closely. This supports the idea that things are normal. The Versus Fits plot shows that the data points are spread out randomly across the range of fitted values. This shows that the data is linear and that the variance is constant. Lastly, the Versus Order plot does not show any patterns that happen over time. This shows that the errors occurred at random times during the experiment, which is an important point. These diagnostic checks make sure that the CM prediction model is accurate. Supplementary Table S13 indicates that the *p*-values for the model, linear interaction, and the individual parameters exceed 0.05, signifying non-significance. The contribution of approximately 2.23% originates from Factor D, 3.56% from Factor E, and 3.26% from Factor B. Factor A and Factor C contribute 24.90% and 2.89%, respectively. The R^2^ value is 86.16%, as indicated in Supplementary Table S13, indicating that the elevated R^2^ value signifies a more accurate alignment between the data and the model. The importance of the correlation is further evidenced by the adjusted R^2^, which stands at 82.14%. The elevated adjusted R^2^ indicates that the proposed mathematical model offers an enhanced understanding of the relationship between the variable and the response. The model’s projected R^2^ reflects an accuracy of 80.08%. Figure 27 illustrates the interaction effect on the compressive modulus with various infill patterns. Supplementary Table S14 presents the regression equation for the compressive modulus across various infill patterns and percentages.

Comparison of Experimental and predicted compressive modulus

**Figure 28:**
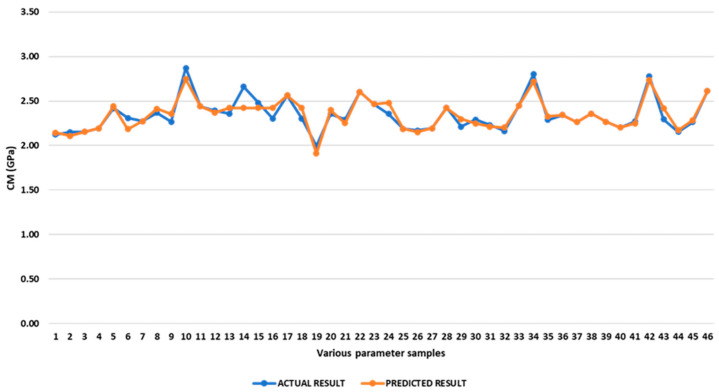
Comparison of experimental and predicted compressive modulus.

Supplementary Table S15 presents both the theoretical and experimental values for the compressive modulus. The results show that the deviations between the anticipated and experimental modulus range from 0% to 5.43% and average 1.79%. We found that maximising responses also gave us the best modulus value. Via optimisation, we found that the inner infill with a rectilinear pattern and a raster angle of 0°, the top infill with a concentric pattern, and the bottom infill with a rectilinear pattern and a raster angle of 45° would result in a compressive modulus of 2.75 GPa. Figure 28 depicts a comparison between the expected and actual compressive modulus for different values of the operating parameters.

### 3.5. Comparison of Mechanical Properties of PETG Specimens

**Figure 29:**
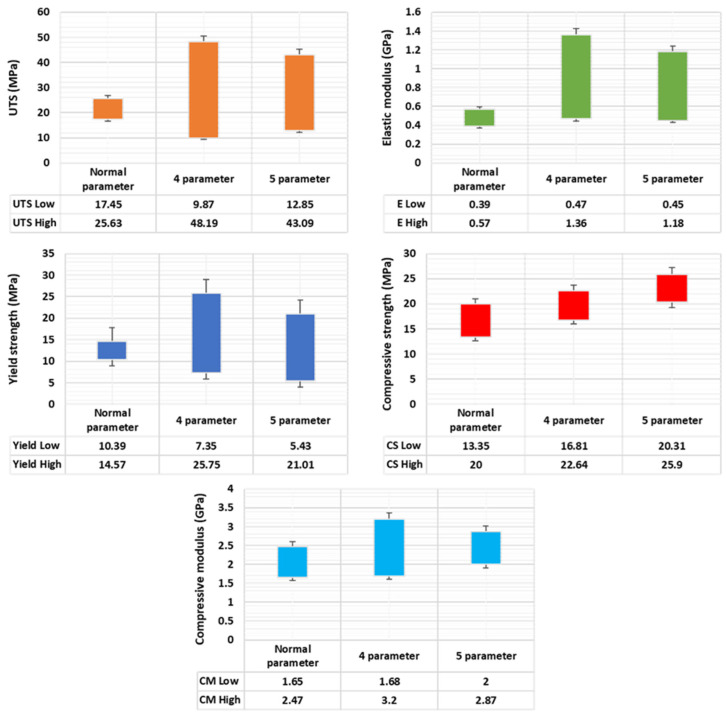
Comparison of mechanical properties of various printing parameters.

The average mechanical properties of the normal parameters are high in previous studies, because in previous studies, specimens were printed with 100% infill density. But, in this research, all specimens were printed with only 50% infill density. Also, we increased the solid layers from 2 to 3. This research work was planned in three stages: (1) normal parameters (the infill pattern and raster angle remains the same in all the layers) [10], (2) four parameters (changing the inner infill pattern, top/bottom infill pattern, inner raster, and top/bottom raster) [11], and (3) five parameters (changing the inner infill pattern, top infill pattern, bottom infill pattern, inner raster, and top/bottom raster). A better comparison of all the mechanical properties of the printed PETG specimens analysed by various parameters (normal, four, and five) is shown in Figure 29. It clearly shows a comparison of the tensile and compressive loads; from this, the four-parameter samples obtain more energy in all mechanical characteristics. Compared with the normal-parameter specimens, the four-parameter specimens’ obtained values are more than 150%. The sample values for the four parameters are likely to be quite near to the value for the five parameters, yet the four parameters still gain a higher level of strength.

In this study, the maximum possibilities to shift the top and bottom patterns involved concentric, rectilinear, and line infill patterns. Also, we selected 0°, 45°, and 90° as the raster angle range. The average mechanical properties of the four- and five-parameter specimens were two times higher than those of the normal parameters. This is due to the structure of the specimens. The maximum UTS obtained from the previous studies (commercial filament) are 35 ± 0.3 MPa with 100% infill density. But the four-parameter specimens’ maximum UTS value of 48.19 MPa is 1.5 times more than that of previous studies. The 0° raster angle relative to the load aligns the applied stress with the strong surfaces, maximising the material’s inherent strength. Non-aligned angles, such as the 90° and 45° orientation, force failure to be governed by the significantly weaker inter-layer adhesion, which is consistent with the lower mechanical values obtained in these directions. For the compressive strength, the five parameters attained more strength compared with other parameters, because the top, bottom, and inner layer variations were helpful in achieving more strength. From this, the four-parameter samples achieved maximum mechanical properties, except for compressive strength. The five-parameter specimens also obtained higher qualities than the normal-parameter specimens, close to the four-parameter specimens. From this comparison, it is understandable that changing the infill pattern of the solid layer and normal layer will help to improve the mechanical properties. Also, if the top and bottom pattern are equally maintained, this will be helpful for achieving high strength. At the same time, the normal-parameter specimens’ properties also increased compared with those in previous studies. Table 4 demonstrates a comparison of the current study with previous studies, with equivalent working conditions. This is due to increasing the solid layer count, reducing the layer thickness to 0.1 mm, and also reducing the printing speed to 30 mm/s. Thus, it is possible to increase the mechanical properties of the PETG material by using 50% infill density with optimisation of the parameters.

## 4. Conclusions

In this research, the mechanical properties of the PETG thermoplastic specimens were analysed by using 50% density and varying the process parameters. A total of 5 parameters combo used to print the specimens. The Design of Experiments approach was performed using Minitab 19.0 software, and an RSM technique was used to develop the mathematical model. The first step of the process was to create a Response Surface Design, using the Box–Behnken Design. Later, mechanical analyses (tensile and compressive) were performed to analyse the properties of PETG. The microstructural behaviour with the mechanical properties of the various-parameter printed PETG specimens were analysed. Using DOE techniques, we determined the individual and interactive effects of various factors that could influence the output results of this study. In this study, the maximum UTS of the PETG specimens obtained was 43.09 MPa, the maximum modulus was 1.18 GPa, and the yield strength was 21.01 MPa. Here, all the maximum properties were obtained via combination of rectilinear and concentric infill patterns, and the lowest values were obtained via combination of line infill patterns. A maximum compressive strength of 25.90 MPa and a maximum compressive modulus of 2.87 GPa were obtained in sample 10. Sample 10 had a combination of rectilinear and concentric infill patterns and a top/bottom raster angle of 45°. In terms of compressive strength, the raster angle is the key parameter that enhances the properties. From these mechanical properties of the various-parameter printed specimens in previous studies [44], the four-parameter specimens’ strength was two times greater than the normal-parameter specimens. Also, these properties were achieved only with 50% infill density. The properties of the four and five parameters were very close, but still the four-parameter specimens’ properties were higher than those of the five parameters. From this, specimens printing with top and bottom layers and with the same infill patterns led to higher strength compared to printing with various infill patterns in the top and bottom layers. The microstructure of the specimen with maximum and minimum strength of all parameters was analysed after the load. It was clearly shown that specimens with fewer voids and smaller cracks obtained more strength. In the rectilinear and concentric infill patterns, the layers were meshed properly, whereas the combination of line infill patterns had more voids and cracks, and the airgap was zero. An ANOVA was performed, the regression equations were created, and the predicted data were found. A Pareto chart was created after the ANOVA; by using the graphs, the major parameters that affected the material quality in each analysis were discovered. The RSM technique indicated that factors were deemed statistically significant when their *p*-values (alpha values) fell below 0.05, while those exceeding 0.05 were regarded as statistically insignificant. The data clearly indicates that the inner raster angle is the most critical factor affecting product strength. From all analyses, the R^2^ value was more than 80%, which shows that the developed model fits the regression model. In addition, the fact that the error percentage between the projected data and the experimental data was less than 5% demonstrates that the regression results are satisfactory.

- In contrast to previous investigations, the standard parameters’ average mechanical characteristics demonstrate a greater magnitude. This discrepancy results from the fact that a 100% infill density was used to manufacture the specimens in previous experiments. It is important to highlight that in this study, all specimens were produced with a uniform infill density of 50%.
- The occurrence of this phenomenon can be traced to the increase in the number of solid layers from two to three. The results of this investigation indicate that the concentric pattern is primarily suitable for bearing tensile load. This conclusion was reached as a result of examination due to the fact that the load was distributed evenly from one end to the other end of the structure.
- In the context of normal-parameter printing, the infill pattern holds significant importance in relation to tensile load. On the other hand, when compressive loads are taken into consideration, the raster angle has a greater impact on the specimen’s strength than the infill pattern does.
- A twofold increase in the average mechanical characteristics was seen in the specimens with four and five parameters as compared to the specimens with normal parameters. This phenomenon can be explained by the structural composition of the specimens.
- Additionally, PETG is an excellent material for the production of parts that are both flexible and have a high resistance to shock. These parts include pressure-clad objects and protective components.
- Due to its heat resistance and good mechanical properties, PETG is used in many sectors such as aerospace components, automotive interior components, bio-based applications, electronic insulators, and medical equipment.
- In future studies, it is strongly recommended to reduce layer thickness from 0.1 mm to 0.05 mm, which will increase the chances of interlayer bonding and the surface finish of the products.
- Further research could focus on leveraging these improved properties to enhance the application range of FDM-printed PETG parts, especially considering its suitability for flexible, shock-resistant, heat-resistant components in sectors like aerospace and medical equipment.

## Figures and Tables

**Table 1:** RSD model for optimised trail using infill patterns and raster angles.

Inner Pattern	Inner Raster Angle (°)	Top Pattern	Bottom Pattern	Top/Bottom Raster Angle (°)
Re	90	Re	Li	45
Re	90	Li	Re	45
Re	90	Re	Re	90
Re	45	Co	Li	45
Li	0	Re	Re	45
Re	90	Co	Re	45
Li	45	Li	Re	45
Re	45	Li	Re	0
Co	90	Re	Re	45
Re	0	Co	Re	45
Co	45	Re	Li	45
Li	45	Re	Re	90
Re	45	Re	Re	45
Re	45	Re	Re	45
Re	45	Re	Re	45
Re	45	Re	Re	45
Re	0	Re	Re	90
Re	45	Re	Re	45
Co	45	Re	Re	90
Re	45	Li	Re	90
Re	45	Re	Co	90
Re	0	Re	Re	0
Co	45	Co	Re	45
Re	45	Co	Re	0
Co	45	Li	Re	45
Re	45	Re	Li	90
Re	90	Re	Re	0
Re	45	Re	Re	45
Co	0	Re	Re	45
Re	45	Re	Co	0
Li	45	Re	Re	0
Re	90	Re	Co	45
Re	45	Co	Co	45
Co	45	Re	Re	0
Re	0	Re	Co	45
Li	45	Re	Co	45
Li	45	Re	Li	45
Re	45	Li	Co	45
Co	45	Re	Co	45
Li	45	Co	Re	45
Re	45	Re	Li	0
Re	0	Li	Re	45
Re	45	Co	Re	90
Li	90	Re	Re	45
Re	0	Re	Li	45
Re	45	Li	Li	45

Rectilinear = Re; Concentric = Co; Lines = Li.

**Table 2:** Average tensile properties of 5 parameters from the printed PETG specimens.

Inner Pattern	Inner Raster Angle (°)	Top Pattern	Bottom Pattern	Top/Bottom Raster Angle (°)	Tensile Strength (MPa)	Young’s Modulus (GPa)	Yield Strength (MPa) (0.2% Offset)
Re	90	Re	Li	45	14.94	0.85	11.35
Re	90	Li	Re	45	14.44	0.79	10.66
Re	90	Re	Re	90	37.06	1.18	21.01
Re	45	Co	Li	45	28.77	0.94	15.73
Li	0	Re	Re	45	15.19	0.90	12.07
Re	90	Co	Re	45	32.57	1.03	18.13
Li	45	Li	Re	45	12.85	0.63	7.96
Re	45	Li	Re	0	14.10	0.61	8.53
Co	90	Re	Re	45	15.18	0.90	12.10
Re	0	Co	Re	45	32.30	0.97	17.10
Co	45	Re	Li	45	14.45	0.79	11.65
Li	45	Re	Re	90	34.95	1.12	20.22
Re	45	Re	Re	45	13.84	0.87	11.16
Re	45	Re	Re	45	14.45	0.88	11.71
Re	45	Re	Re	45	13.88	0.85	11.15
Re	45	Re	Re	45	13.77	0.90	11.56
Re	0	Re	Re	90	38.44	1.18	20.90
Re	45	Re	Re	45	13.68	0.83	9.37
Co	45	Re	Re	90	38.03	1.14	19.82
Re	45	Li	Re	90	39.26	1.12	19.69
Re	45	Re	Co	90	43.09	1.14	20.90
Re	0	Re	Re	0	14.40	0.51	7.15
Co	45	Co	Re	45	30.17	0.95	15.30
Re	45	Co	Re	0	26.32	0.81	13.78
Co	45	Li	Re	45	12.94	0.65	7.83
Re	45	Re	Li	90	40.63	1.06	18.10
Re	90	Re	Re	0	14.40	0.54	7.54
Re	45	Re	Re	45	14.04	0.79	9.46
Co	0	Re	Re	45	14.16	0.80	9.79
Re	45	Re	Co	0	27.81	0.84	13.64
Li	45	Re	Re	0	13.52	0.50	7.55
Re	90	Re	Co	45	29.24	0.85	12.06
Re	45	Co	Co	45	34.94	1.04	17.78
Co	45	Re	Re	0	14.13	0.52	7.65
Re	0	Re	Co	45	30.89	0.89	14.95
Li	45	Re	Co	45	31.27	0.80	12.31
Li	45	Re	Li	45	17.76	0.67	13.73
Re	45	Li	Co	45	29.47	0.73	11.00
Co	45	Re	Co	45	29.81	0.74	10.14
Li	45	Co	Re	45	26.94	0.83	12.96
Re	45	Re	Li	0	25.23	0.82	11.94
Re	0	Li	Re	45	14.57	0.60	8.00
Re	45	Co	Re	90	40.16	0.89	15.31
Li	90	Re	Re	45	13.92	0.67	8.53
Re	0	Re	Li	45	17.73	0.60	7.50
Re	45	Li	Li	45	14.77	0.45	5.43

Re = Rectilinear; Li = Lines; Co = Concentric.

**Table 3:** Average compressive properties of 5 parameters from the printed PETG specimens.

Inner Pattern	Inner Raster Angle (°)	Top Pattern	Bottom Pattern	Top/ Bottom Raster Angle (°)	Compressive Strength (MPa)	Compressive Modulus (GPa)
Re	90	Re	Li	45	21.86	2.12
Re	90	Li	Re	45	21.35	2.15
Re	90	Re	Re	90	21.22	2.15
Re	45	Co	Li	45	20.31	2.19
Li	0	Re	Re	45	20.69	2.42
Re	90	Co	Re	45	22.10	2.31
Li	45	Li	Re	45	21.96	2.27
Re	45	Li	Re	0	21.32	2.37
Co	90	Re	Re	45	22.49	2.27
Re	0	Co	Re	45	25.90	2.87
Co	45	Re	Li	45	23.09	2.44
Li	45	Re	Re	90	21.92	2.39
Re	45	Re	Re	45	22.53	2.36
Re	45	Re	Re	45	22.24	2.66
Re	45	Re	Re	45	22.65	2.48
Re	45	Re	Re	45	22.42	2.30
Re	0	Re	Re	90	23.55	2.56
Re	45	Re	Re	45	22.46	2.30
Co	45	Re	Re	90	22.27	2.00
Re	45	Li	Re	90	21.72	2.35
Re	45	Re	Co	90	23.11	2.29
Re	0	Re	Re	0	22.69	2.60
Co	45	Co	Re	45	23.72	2.46
Re	45	Co	Re	0	23.31	2.35
Co	45	Li	Re	45	23.37	2.19
Re	45	Re	Li	90	21.77	2.17
Re	90	Re	Re	0	20.56	2.19
Re	45	Re	Re	45	22.48	2.43
Co	0	Re	Re	45	21.90	2.21
Re	45	Re	Co	0	21.94	2.29
Li	45	Re	Re	0	21.02	2.23
Re	90	Re	Co	45	22.43	2.16
Re	45	Co	Co	45	24.28	2.45
Co	45	Re	Re	0	24.91	2.80
Re	0	Re	Co	45	21.35	2.29
Li	45	Re	Co	45	22.81	2.34
Li	45	Re	Li	45	22.03	2.27
Re	45	Li	Co	45	23.56	2.36
Co	45	Re	Co	45	23.11	2.27
Li	45	Co	Re	45	22.12	2.20
Re	45	Re	Li	0	21.75	2.27
Re	0	Li	Re	45	24.76	2.78
Re	45	Co	Re	90	22.29	2.29
Li	90	Re	Re	45	21.29	2.15
Re	0	Re	Li	45	22.46	2.27
Re	45	Li	Li	45	23.57	2.61

Re = Rectilinear; Li = Lines; Co = Concentric.

**Table 4:** Comparison of mechanical properties under equivalent conditions.

Study	Material	Infill Density	UTS	CS	Relative Improvement
Current Study	PETG	50%	43.09 MPa	25.90 MPa	-
Faidallah et al. [54]	PETG	100%	42.0 MPa	N/A	+51.26% UTS
Martins et al. [55]	PETG	50%	33 MPa	N/A	+23.41% UTS
Lacob et al. [56]	PETG	100	43.24 MPa	30.33	+49.82% UTS and 41.44% CS
Lakshman et al. [57]	PETG	100%	44.5 MPa	N/A	+48.36% UTS

N/A = Not Available.

## Data Availability

The original contributions presented in this study are included in the article/Supplementary Material. Further inquiries can be directed to the corresponding authors.

## Supplementary Materials

The following supporting information can be downloaded at https://www.mdpi.com/article/10.3390/polym17233175/s1, Table S1: Analysis of variance of ultimate tensile strength; Table S2: Regression equation of ultimate tensile strength with various infill patterns and infill percentage; Table S3: Experimental and mathematically predicted ultimate tensile strength; Table S4: Analysis of variance of elastic modulus; Table S5: Regression equation of elastic modulus with various infill patterns and infill percentage; Table S6: Experimental and mathematically predicted elastic modulus; Table S7: Analysis of variance of yield strength; Table S8: Regression equation of yield strength with various infill patterns and infill percentage; Table S9: Experimental and mathematically predicted yield strength; Table S10: Analysis of variance of compressive strength; Table S11: Regression equation of compressive strength with various infill patterns and infill percentage; Table S12: Experimental and mathematically predicted compressive strength; Table S13: Analysis of variance of compressive modulus; Table S14: Regression equation of compressive modulus with various infill patterns and infill percentage; Table S15: Experimental and mathematically predicted compressive modulus.

## References

[1] Bello K.A., Maladzhi R.W. (2025). Innovative and Best Practices in Sustainable Strategies for Waste Reduction in Additive Manufacturing. Hybrid Adv..

[2] Yang J., Li B., Liu J., Tu Z., Wu X. (2024). Application of Additive Manufacturing in the Automobile Industry: A Mini Review. Processes.

[3] Zhou L., Miller J., Vezza J., Mayster M., Raffay M., Justice Q., Al Tamimi Z., Hansotte G., Sunkara L.D., Bernat J. (2024). Additive Manufacturing: A Comprehensive Review. Sensors.

[4] Ng W.L., Goh G.L., Goh G.D., Ten J.S.J., Yeong W.Y. (2024). Progress and Opportunities for Machine Learning in Materials and Processes of Additive Manufacturing. Adv. Mater..

[5] Karkaria V., Goeckner A., Zha R., Chen J., Zhang J., Zhu Q., Cao J., Gao R.X., Chen W. (2024). Towards a Digital Twin Framework in Additive Manufacturing: Machine Learning and Bayesian Optimization for Time Series Process Optimization. J. Manuf. Syst..

[6] Bănică C.F., Sover A., Anghel D.C. (2024). Printing the Future Layer by Layer: A Comprehensive Exploration of Additive Manufacturing in the Era of Industry 4.0. Appl. Sci..

[7] Yang Y., Jiang R., Han C., Chen J., Li H., Wang Y., Tang J., Zhou H., Hu W., Zheng B. (2024). Frontiers in Laser Additive Manufacturing Technology. Addit. Manuf. Front..

[8] Liu X., Erkoyuncu J.A., Fuh J.Y.H., Lu W.F., Li B. (2025). Knowledge Extraction for Additive Manufacturing Process via Named Entity Recognition with LLMs. Robot. Comput. Integr. Manuf..

[9] Kumaresan R., Kulandaivel S., Samykano M., Keng N.W., Sharuzi Wan Harun W., Mat Noor M., Badadhe A.M., Pahang Al-Sultan Abdullah M., Tun Razak L. (2026). Additive Manufacturing: An Overview of Printing Technologies. J. Adv. Res. Appl. Sci. Eng. Technol. J..

[10] Pelin G., Sonmez M., Pelin C.E. (2024). The Use of Additive Manufacturing Techniques in the Development of Polymeric Molds: A Review. Polymers.

[11] Samykano M., Kumaresan R., Kananathan J., Kadirgama K., Pandey A.K. (2024). An Overview of Fused Filament Fabrication Technology and the Advancement in PLA-Biocomposites. Int. J. Adv. Manuf. Technol..

[12] Dubey D., Singh S.P., Behera B.K. (2024). A Review on Recent Advancements in Additive Manufacturing Techniques. Proc. Inst. Mech. Eng. Part E J. Process Mech. Eng..

[13] Divakaran N., Das J.P., PV A.K., Mohanty S., Ramadoss A., Nayak S.K. (2022). Comprehensive Review on Various Additive Manufacturing Techniques and Its Implementation in Electronic Devices. J. Manuf. Syst..

[14] Roscoe S., Cousins P.D., Handfield R. (2023). Transitioning Additive Manufacturing from Rapid Prototyping to High-Volume Production: A Case Study of Complex Final Products. J. Prod. Innov. Manag..

[15] Patel S., Liu Y., Siddique Z., Ghamarian I. (2024). Metal Additive Manufacturing: Principles and Applications. J. Manuf. Process..

[16] Srivastava M., Jayakumar V., Udayan Y., Sathishkumar M., Muthu S.M., Gautam P., Nag A. (2024). Additive Manufacturing of Titanium Alloy for Aerospace Applications: Insights into the Process, Microstructure, and Mechanical Properties. Appl. Mater. Today.

[17] Paul A.A., Aladese A.D., Marks R.S. (2024). Additive Manufacturing Applications in Biosensors Technologies. Biosensors.

[18] Sarıkaya M., Başcıl Önler D., Dağlı S., Hartomacıoğlu S., Günay M., Królczyk G.M. (2024). A Review on Aluminum Alloys Produced by Wire Arc Additive Manufacturing (WAAM): Applications, Benefits, Challenges and Future Trends. J. Mater. Res. Technol..

[19] Amaya-Rivas J.L., Perero B.S., Helguero C.G., Hurel J.L., Peralta J.M., Flores F.A., Alvarado J.D. (2024). Future Trends of Additive Manufacturing in Medical Applications: An Overview. Heliyon.

[20] Hu X., Jiang H., Liu Z., Gao M., Liu G., Tian S., Zeng F. (2025). The Global Potato-Processing Industry: A Review of Production, Products, Quality and Sustainability. Foods.

[21] Sasi A., Srivastava M., Dash K. (2025). Role of Additive Manufacturing in Developing Functionally Graded Materials for Nuclear Applications. Front. Nucl. Eng..

[22] Iqbal H., Fernandes Q., Idoudi S., Basineni R., Billa N. (2024). Status of Polymer Fused Deposition Modeling (FDM)-Based Three-Dimensional Printing (3DP) in the Pharmaceutical Industry. Polymers.

[23] Wang Z., Wang L., Tang F., Chen J. (2023). Multi-Material Additive Manufacturing via Fused Deposition Modeling 3D Printing: A Systematic Review on the Material Feeding Mechanism. Proc. Inst. Mech. Eng. Part E J. Process Mech. Eng..

[24] Siemiński P. (2021). Introduction to Fused Deposition Modeling. Additive Manufacturing.

[25] Zhang J., Li D., Wang M. (2024). Multi-Material Fused Deposition Modelling of Structural–Functional Integrated Absorber with Multi-Scale Structure Possessing Tunable Broadband Microwave Absorption. Mater. Des..

[26] Nguyen K.Q., Vuillaume P.Y., Hu L., Vachon A., Diouf-Lewis A., Marcoux P.L., Robert M., Elkoun S. (2024). Effect of in Situ Thermal Treatment on Interlayer Adhesion of 3D Printed Polyetherimide (PEI) Parts Produced by Fused Deposition Modeling (FDM). Mater. Today Commun..

[27] Makki T., Vattathurvalappil S.H., Theravalappil R., Nazir A., Alhajeri A., Azeem M.A., Mahdi E., Ummer A.C., Ali U. (2024). 3D and 4D Printing: A Review of Virgin Polymers Used in Fused Deposition Modeling. Compos. Part C Open Access.

[28] Franco Urquiza E.A. (2024). Advances in Additive Manufacturing of Polymer-Fused Deposition Modeling on Textiles: From 3D Printing to Innovative 4D Printing—A Review. Polymers.

[29] Kumar A., Dixit A.R., Sreenivasa S. (2024). Mechanical Properties of Additively Manufactured Polymeric Composites Using Sheet Lamination Technique and Fused Deposition Modeling: A Review. Polym. Adv. Technol..

[30] Khan I., Tariq M., Abas M., Shakeel M., Hira F., Al Rashid A., Koç M. (2023). Parametric Investigation and Optimisation of Mechanical Properties of Thick Tri-Material Based Composite of PLA-PETG-ABS 3D-Printed Using Fused Filament Fabrication. Compos. Part C Open Access.

[31] Syrlybayev D., Zharylkassyn B., Seisekulova A., Akhmetov M., Perveen A., Talamona D. (2021). Optimisation of Strength Properties of FDM Printed Parts—A Critical Review. Polymers.

[32] Wikło M., Byczuk B.H., Skrzek K. (2025). Mechanical Characterization of FDM 3D-Printed Components Using Advanced Measurement and Modeling Techniques. Materials.

[33] Muzli M.F., Ismail K.I., Yap T.C. (2024). Effects of Infill Density and Printing Speed on The Tensile Behaviour of Fused Deposition Modelling 3D Printed PLA Specimens. J. Eng. Technol. Appl. Phys..

[34] Valvez S., Silva A.P., Reis P.N.B. (2022). Optimization of Printing Parameters to Maximize the Mechanical Properties of 3D-Printed PETG-Based Parts. Polymers.

[35] Loskot J., Jezbera D., Loskot R., Bušovský D., Barylski A., Glowka K., Duda P., Aniołek K., Voglová K., Zubko M. (2023). Influence of Print Speed on the Microstructure, Morphology, and Mechanical Properties of 3D-Printed PETG Products. Polym. Test..

[36] Kumaresan R., Samykano M., Kadirgama K., Pandey A.K., Rahman M.M. (2023). Effects of Printing Parameters on the Mechanical Characteristics and Mathematical Modeling of FDM-Printed PETG. Int. J. Adv. Manuf. Technol..

[37] Chahdoura S., Bahloul R., Tlija M., Tahan A. (2025). Multi-Objective Optimization of PLA-FDM Parameters for Enhancement of Industrial Product Mechanical Performance Based on GRA-RSM and BBD. Prog. Addit. Manuf..

[38] Rivera-López F., Pavón M.M.L., Correa E.C., Molina M.H. (2024). Effects of Nozzle Temperature on Mechanical Properties of Polylactic Acid Specimens Fabricated by Fused Deposition Modeling. Polymers.

[39] Mencarelli M., Sisella M., Puggelli L., Innocenti B., Volpe Y. (2025). Sensitivity Analysis of 3D Printing Parameters on Mechanical Properties of Fused Deposition Modeling-Printed Polylactic Acid Parts. Appl. Mech..

[40] Ulkir O., Karadag A. (2025). Prediction and Optimization of PETG Part Hardness in 3D Printing: A Comparative Study of Experimental Design Methods. Polym. Adv. Technol..

[41] Firtikiadis L., Tzotzis A., Kyratsis P., Efkolidis N. (2024). Response Surface Methodology (RSM)-Based Evaluation of the 3D-Printed Recycled-PETG Tensile Strength. Appl. Mech..

[42] Tunçel O., Kahya Ç., Tüfekci K. (2024). Optimization of Flexural Performance of PETG Samples Produced by Fused Filament Fabrication with Response Surface Method. Polymers.

[43] Kumaresan R., Kadirgama K., Samykano M., Harun W.S.W., Thirugnanasambandam A., Kanny K. (2025). In-Depth Study and Optimization of Process Parameters to Enhance Tensile and Compressive Strengths of PETG in FDM Technology. J. Mater. Res. Technol..

[44] Kumaresan R., Kadirgama K., Samykano M., Harun W.S.W., Thirugnanasambandam A., Aslfattahi N., Samylingam L., Kok C.K., ghazali M.f. (2025). Optimization of Inter-Layer Printing Parameters for Enhanced Mechanical Performance of PETG in Fused Deposition Modeling (FDM). Results Eng..

[45] Zhou X., Hsieh S.J., Ting C.C. (2018). Modelling and Estimation of Tensile Behaviour of Polylactic Acid Parts Manufactured by Fused Deposition Modelling Using Finite Element Analysis and Knowledge-Based Library. Virtual Phys. Prototyp..

[46] Webbe Kerekes T., Lim H., Joe W.Y., Yun G.J. (2019). Characterization of Process–Deformation/Damage Property Relationship of Fused Deposition Modeling (FDM) 3D-Printed Specimens. Addit. Manuf..

[47] Omer M.A.E., Shaban I.A., Mourad A.H., Hegab H. (2025). Advances in Interlayer Bonding in Fused Deposition Modelling: A Comprehensive Review. Virtual Phys. Prototyp..

[48] Melentiev R., Lagerweij A., Lubineau G. (2024). Multiprocess Additive Manufacturing via Fused Deposition Modeling, Chemical Deposition, and Electroplating with Tough Interfacial Adhesion. Smart Mater. Manuf..

[49] Bakhtiari H., Aamir M., Tolouei-Rad M. (2023). Effect of 3D Printing Parameters on the Fatigue Properties of Parts Manufactured by Fused Filament Fabrication: A Review. Appl. Sci..

[50] Yang Y., Yang M., He C., Qi F., Wang D., Peng S., Shuai C. (2021). Rare Earth Improves Strength and Creep Resistance of Additively Manufactured Zn Implants. Compos. Part B Eng..

[51] Houriet C., Ulyanov B., Pascoe J.A., Masania K. (2025). Wood-Inspired Interlocking Junctions Using 3D-Printed Liquid Crystal Polymers. Addit. Manuf..

[52] Guessasma S., Belhabib S., Nouri H., Ben Hassana O. (2016). Anisotropic Damage Inferred to 3D Printed Polymers Using Fused Deposition Modelling and Subject to Severe Compression. Eur. Polym. J..

[53] Domínguez-Rodríguez G., Ku-Herrera J.J., Hernández-Pérez A. (2018). An Assessment of the Effect of Printing Orientation, Density, and Filler Pattern on the Compressive Performance of 3D Printed ABS Structures by Fuse Deposition. Int. J. Adv. Manuf. Technol..

[54] Faidallah R.F., Hanon M.M., Szakál Z., Oldal I. (2025). Mechanical Characterization of 3D-Printed Carbon Fiber-Reinforced Polymer Composites and Pure Polymers: Tensile and Compressive Behavior Analysis. Int. Rev. Appl. Sci. Eng..

[55] Martins R.F., Branco R., Martins M., Macek W., Marciniak Z., Silva R., Trindade D., Moura C., Franco M., Malça C. (2024). Mechanical Properties of Additively Manufactured Polymeric Materials—PLA and PETG—For Biomechanical Applications. Polymers.

[56] Iacob D.V., Zisopol D.G., Minescu M. (2024). Technical-Economical Study on the Optimization of FDM Parameters for the Manufacture of PETG and ASA Parts. Polymers.

[57] Lakshman S.S.V., Karthick A., Dinesh C. (2024). Evaluation of Mechanical Properties of 3D Printed PETG and Polyamide (6) Polymers. Chem. Phys. Impact.

