# Supplementary material for: Advanced RSM-Driven Optimisation for Enhancing the Mechanical Performance of FDM-Printed PETG: A Correlated Microstructural and Mechanical Property Investigation
Source: Polymers (Basel). 2025 Nov 29;17(23):3175. doi: 10.3390/polym17233175 (PMC12694470; doi:10.3390/polym17233175)
Supplement: Supplementary file 1 [file polymers-17-03175-s001.zip › polymers-3983632-supplementary.pdf]

Supplementary file

# Advanced RSM-Driven Optimization for Enhancing Mechanical Performance of FDM-Printed PETG: A Correlated Microstructural and Mechanical Property Investigation

Table S1. Analysis of variance of ultimate tensile strength.

| Source | DF | Contribution | Adj SS | Adj MS | F-Value | P-Value |
|---|---|---|---|---|---|---|
| Model | 35 | 96.63% | 4416.68 | 126.191 | 8.20 | 0.001 |
| Linear | 8 | 70.00% | 727.63 | 90.954 | 5.91 | 0.006 |
| A | 1 | 0.05% | 0.29 | 0.287 | 0.02 | 0.894 |
| B | 1 | 35.76% | 96.24 | 96.236 | 6.25 | 0.031 |
| C | 2 | 3.11% | 1.24 | 0.620 | 0.04 | 0.961 |
| D | 2 | 14.96% | 224.34 | 112.172 | 7.29 | 0.011 |
| E | 2 | 16.12% | 308.27 | 154.133 | 10.02 | 0.004 |
| Square | 2 | 22.91% | 877.03 | 438.513 | 28.49 | 0.000 |
| A*A | 1 | 0.44% | 13.35 | 13.349 | 0.87 | 0.374 |
| B*B | 1 | 22.47% | 790.65 | 790.648 | 51.38 | 0.000 |
| 2-Way Interaction | 25 | 3.73% | 170.29 | 6.812 | 0.44 | 0.952 |
| A*B | 1 | 0.01% | 0.47 | 0.472 | 0.03 | 0.864 |
| A*C | 2 | 0.04% | 1.47 | 0.734 | 0.05 | 0.954 |
| A*D | 2 | 0.03% | 0.33 | 0.167 | 0.01 | 0.989 |
| A*E | 2 | 0.03% | 1.48 | 0.742 | 0.05 | 0.953 |
| B*C | 2 | 0.21% | 1.77 | 0.883 | 0.06 | 0.945 |
| B*D | 2 | 0.70% | 39.47 | 19.737 | 1.28 | 0.319 |
| B*E | 2 | 0.70% | 32.08 | 16.038 | 1.04 | 0.388 |
| C*D | 4 | 0.43% | 10.64 | 2.661 | 0.17 | 0.947 |
| C*E | 4 | 0.84% | 35.87 | 8.968 | 0.58 | 0.682 |
| D*E | 4 | 0.73% | 33.26 | 8.316 | 0.54 | 0.710 |
| Error | 10 | 3.37% | 153.90 | 15.390 | | |
| Lack-of-Fit | 5 | 3.36% | 153.52 | 30.704 | 409.14 | 0.000 |
| Pure Error | 5 | 0.01% | 0.38 | 0.075 | | |
| Total | 45 | 100.00% | | | | |
| $R^2$ = 96.63% | | | | | | |
| $R^2$ - adjusted = 89.69% | | | | | | |
| $R^2$ - predicted = 84.85 % | | | | | | |

Factors: A=Inner raster, B= T/B raster, C= Inner pattern, D= Top pattern, E= Bottom pattern

Table S2. Regression equation of ultimate tensile strength with various infill patterns and infill percentage.

| Inner pattern | Top pattern | Bottom pattern | Regression equation |
|---|---|---|---|
| Co | Co | Co | 49.2 - 0.043 A - 0.399 B + 0.000689 A * A + 0.005303 B* B - 0.000170 A * B |

| | | | |
|---|---|---|---|
| Li | Co | Co | 50.8 - 0.068 A - 0.427 B + 0.000689 A * A + 0.005303 B*B - 0.000170 A*B |
| Re | Co | Co | 44.19 - 0.062 A - 0.406 B + 0.000689 A* A + 0.005303 B*B - 0.000170 A*B |
| Co | Li | Co | 33.3 - 0.047 A - 0.274 B + 0.000689 A* A + 0.005303 B*B - 0.000170 A*B |
| Li | Li | Co | 38.0 - 0.073 A - 0.301 B + 0.000689 A* A + 0.005303 B*B - 0.000170 A*B |
| Re | Li | Co | 33.26 - 0.066 A - 0.280 B + 0.000689 A* A + 0.005303 B*B - 0.000170 A*B |
| Co | Re | Co | 33.65 - 0.054 A - 0.294 B + 0.000689 A* A + 0.005303 B*B - 0.000170 A*B |
| Li | Re | Co | 37.49 - 0.079 A - 0.321 B + 0.000689 A* A + 0.005303 B*B - 0.000170 A*B |
| Re | Re | Co | 31.66 - 0.073 A - 0.300 B + 0.000689 A* A + 0.005303 B*B - 0.000170 A*B |
| Co | Co | Li | 36.3 - 0.056 A - 0.398 B + 0.000689 A* A + 0.005303 B*B - 0.000170 A*B |
| Li | Co | Li | 39.7 - 0.081 A - 0.426 B + 0.000689 A* A + 0.005303 B*B - 0.000170 A*B |
| Re | Co | Li | 38.52 - 0.075 A - 0.404 B + 0.000689 A* A + 0.005303 B*B - 0.000170 A*B |
| Co | Li | Li | 11.9 - 0.060 A - 0.272 B + 0.000689 A* A + 0.005303 B*B - 0.000170 A*B |
| Li | Li | Li | 18.4 - 0.085 A - 0.300 B + 0.000689 A* A + 0.005303 B*B - 0.000170 A*B |
| Re | Li | Li | 19.07 - 0.079 A - 0.278 B + 0.000689 A* A + 0.005303 B*B - 0.000170 A*B |
| Co | Re | Li | 18.80 - 0.066 A - 0.292 B + 0.000689 A* A + 0.005303 B*B - 0.000170 A*B |
| Li | Re | Li | 24.48 - 0.092 A - 0.320 B + 0.000689 A* A + 0.005303 B*B - 0.000170 A*B |
| Re | Re | Li | 24.05 - 0.085 A - 0.298 B + 0.000689 A* A + 0.005303 B*B - 0.000170 A*B |
| Co | Co | Re | 33.78 - 0.032 A - 0.310 B + 0.000689 A* A + 0.005303 B*B - 0.000170 A*B |
| Li | Co | Re | 32.92 - 0.058 A - 0.337 B + 0.000689 A* A + 0.005303 B*B - 0.000170 A*B |
| Re | Co | Re | 31.50 - 0.051 A - 0.316 B + 0.000689 A* A + 0.005303 B*B - 0.000170 A*B |
| Co | Li | Re | 11.08 - 0.037 A - 0.184 B + 0.000689 A* A + 0.005303 B*B - 0.000170 A*B |
| Li | Li | Re | 13.38 - 0.062 A - 0.211 B + 0.000689 A* A + 0.005303 B*B - 0.000170 A*B |
| Re | Li | Re | 13.81 - 0.056 A - 0.190 B + 0.000689 A* A + 0.005303 B*B - 0.000170 A*B |
| Co | Re | Re | 13.64 - 0.043 A - 0.204 B + 0.000689 A* A + 0.005303 B*B - 0.000170 A*B |
| Li | Re | Re | 15.04 - 0.068 A - 0.232 B + 0.000689 A* A + 0.005303 B*B - 0.000170 A*B |

| | | | |
|---|---|---|---|
| Re | Re | Re | 14.40 - 0.0621 A - 0.2101 B + 0.000689 A* A + 0.005303 B*B - 0.000170 A*B |

Re = Rectilinear, Li = Lines, Co = Concentric

Table S3. Experimental and mathematically predicted ultimate tensile strength.

| **Inner pattern** | **Inner raster (°)** | **Top pattern** | **Bottom pattern** | **Top/ bottom raster** | **Experimental result of UTS (MPa)** | **Predicted result of UTS (MPa)** | **Percentage of error (%)** |
|---|---|---|---|---|---|---|---|
| Re | 90 | Re | Li | 45 | 14.94 | 18.57 | 24.26 |
| Re | 90 | Li | Re | 45 | 14.44 | 15.86 | 9.81 |
| Re | 90 | Re | Re | 90 | 37.06 | 37.06 | 0.00 |
| Re | 45 | Co | Li | 45 | 28.77 | 28.77 | 0.00 |
| Li | 0 | Re | Re | 45 | 15.19 | 15.36 | 1.11 |
| Re | 90 | Co | Re | 45 | 32.57 | 28.30 | 13.10 |
| Li | 45 | Li | Re | 45 | 12.85 | 12.85 | 0.00 |
| Re | 45 | Li | Re | 0 | 14.10 | 12.69 | 10.05 |
| Co | 90 | Re | Re | 45 | 15.18 | 16.21 | 6.83 |
| Re | 0 | Co | Re | 45 | 32.30 | 28.03 | 13.21 |
| Co | 45 | Re | Li | 45 | 14.45 | 14.45 | 0.00 |
| Li | 45 | Re | Re | 90 | 34.95 | 34.78 | 0.48 |
| Re | 45 | Re | Re | 45 | 13.84 | 13.94 | 0.73 |
| Re | 45 | Re | Re | 45 | 14.45 | 13.94 | 3.48 |
| Re | 45 | Re | Re | 45 | 13.88 | 13.94 | 0.49 |
| Re | 45 | Re | Re | 45 | 13.77 | 13.94 | 1.23 |
| Re | 0 | Re | Re | 90 | 38.44 | 38.44 | 0.00 |
| Re | 45 | Re | Re | 45 | 13.68 | 13.94 | 1.92 |
| Co | 45 | Re | Re | 90 | 38.03 | 37.00 | 2.72 |
| Re | 45 | Li | Re | 90 | 39.26 | 37.85 | 3.61 |
| Re | 45 | Re | Co | 90 | 43.09 | 45.07 | 4.59 |
| Re | 0 | Re | Re | 0 | 14.40 | 14.40 | 0.00 |
| Co | 45 | Co | Re | 45 | 30.17 | 30.17 | 0.00 |
| Re | 45 | Co | Re | 0 | 26.32 | 30.59 | 16.21 |
| Co | 45 | Li | Re | 45 | 12.94 | 12.94 | 0.00 |
| Re | 45 | Re | Li | 90 | 40.63 | 37.01 | 8.92 |
| Re | 90 | Re | Re | 0 | 14.40 | 14.40 | 0.00 |
| Re | 45 | Re | Re | 45 | 14.04 | 13.94 | 0.70 |
| Co | 0 | Re | Re | 45 | 14.16 | 15.19 | 7.32 |
| Re | 45 | Re | Co | 0 | 27.81 | 29.79 | 7.12 |
| Li | 45 | Re | Re | 0 | 13.52 | 13.35 | 1.24 |
| Re | 90 | Re | Co | 45 | 29.24 | 27.26 | 6.77 |
| Re | 45 | Co | Co | 45 | 34.94 | 34.94 | 0.00 |
| Co | 45 | Re | Re | 0 | 14.13 | 13.10 | 7.33 |
| Re | 0 | Re | Co | 45 | 30.89 | 28.91 | 6.41 |
| Li | 45 | Re | Co | 45 | 31.27 | 31.27 | 0.00 |
| Li | 45 | Re | Li | 45 | 17.76 | 17.76 | 0.00 |
| Re | 45 | Li | Co | 45 | 29.47 | 29.47 | 0.00 |
| Co | 45 | Re | Co | 45 | 29.81 | 29.81 | 0.00 |
| Li | 45 | Co | Re | 45 | 26.94 | 26.94 | 0.00 |

| | | | | | | | |
|---|---|---|---|---|---|---|---|
| Re | 45 | Re | Li | 0 | 25.23 | 21.60 | 14.37 |
| Re | 0 | Li | Re | 45 | 14.57 | 15.99 | 9.72 |
| Re | 45 | Co | Re | 90 | 40.16 | 44.43 | 10.63 |
| Li | 90 | Re | Re | 45 | 13.92 | 14.09 | 1.21 |
| Re | 0 | Re | Li | 45 | 17.73 | 21.36 | 20.44 |
| Re | 45 | Li | Li | 45 | 14.77 | 14.77 | 0.00 |
| Total error % | | | | | | | 4.70% |

Re = Rectilinear, Li = Lines, Co = Concentric

Table S4. Analysis of variance of elastic modulus.

| Source | DF | Contribution | Adj SS | Adj MS | F-Value | P-Value |
|---|---|---|---|---|---|---|
| Model | 35 | 94.14% | 1.54326 | 0.044093 | 4.59 | 0.007 |
| Linear | 8 | 71.77% | 0.21616 | 0.027020 | 2.81 | 0.064 |
| A | 1 | 0.50% | 0.00449 | 0.004488 | 0.47 | 0.510 |
| B | 1 | 51.29% | 0.00773 | 0.007734 | 0.80 | 0.391 |
| C | 2 | 3.17% | 0.03539 | 0.017694 | 1.84 | 0.209 |
| D | 2 | 13.86% | 0.16983 | 0.084914 | 8.83 | 0.006 |
| E | 2 | 2.94% | 0.03172 | 0.015862 | 1.65 | 0.240 |
| Square | 2 | 1.61% | 0.02150 | 0.010750 | 1.12 | 0.365 |
| A*A | 1 | 0.34% | 0.00673 | 0.006732 | 0.70 | 0.422 |
| B*B | 1 | 1.27% | 0.00557 | 0.005570 | 0.58 | 0.464 |
| 2-Way Interaction | 25 | 20.75% | 0.34024 | 0.013610 | 1.42 | 0.290 |
| A*B | 1 | 0.01% | 0.00017 | 0.000169 | 0.02 | 0.897 |
| A*C | 2 | 2.65% | 0.03063 | 0.015313 | 1.59 | 0.251 |
| A*D | 2 | 0.45% | 0.01101 | 0.005504 | 0.57 | 0.582 |
| A*E | 2 | 1.65% | 0.02700 | 0.013501 | 1.40 | 0.290 |
| B*C | 2 | 2.04% | 0.00066 | 0.000328 | 0.03 | 0.967 |
| B*D | 2 | 3.97% | 0.11089 | 0.055445 | 5.77 | 0.022 |
| B*E | 2 | 4.46% | 0.07304 | 0.036520 | 3.80 | 0.059 |
| C*D | 4 | 0.21% | 0.01386 | 0.003466 | 0.36 | 0.831 |
| C*E | 4 | 1.21% | 0.02395 | 0.005989 | 0.62 | 0.657 |
| D*E | 4 | 4.10% | 0.06724 | 0.016810 | 1.75 | 0.216 |
| Error | 10 | 5.86% | 0.09614 | 0.009614 | | |
| Lack-of-Fit | 5 | 5.41% | 0.08868 | 0.017737 | 11.89 | 0.008 |
| Pure Error | 5 | 0.46% | 0.00746 | 0.001492 | | |
| Total | 45 | 100.00% | | | | |
| R2 = 94.14% | | | | | | |
| R2 - adjusted = 87.72% | | | | | | |
| R2 - predicted = 82.16 % | | | | | | |

Factors: A=Inner raster, B= T/B raster, C= Inner pattern, D= Top pattern, E= Bottom pattern

Table S5. Regression equation of elastic modulus with various infill patterns and infill percentage.

| Inner pattern | Top pattern | Bottom pattern | Regression equation |
|---|---|---|---|
| Co | Co | Co | 0.972 + 0.00263 A - 0.00452 B- 0.000015 A*A + 0.000014 B*B - 0.000003 A*B |
| Li | Co | Co | 1.136 - 0.00109 A - 0.00459 B - 0.000015 A*A + 0.000014 B*B - 0.000003 A*B |
| Re | Co | Co | 1.165 + 0.00159 A - 0.00416 B - 0.000015 A*A + 0.000014 B*B - 0.000003 A*B |
| Co | Li | Co | 0.231 + 0.00414 A + 0.00021 B - 0.000015 A*A + 0.000014 B*B - 0.000003 A*B |
| Li | Li | Co | 0.496 + 0.00043 A + 0.00014 B - 0.000015 A*A + 0.000014 B*B - 0.000003 A*B |
| Re | Li | Co | 0.578 + 0.00310 A + 0.00058 B - 0.000015 A*A + 0.000014 B*B - 0.000003 A*B |
| Co | Re | Co | 0.566 + 0.00213 A + 0.00187 B - 0.000015 A*A + 0.000014 B*B - 0.000003 A*B |
| Li | Re | Co | 0.800 - 0.00159 A + 0.00180 B - 0.000015 A*A + 0.000014 B*B - 0.000003 A*B |
| Re | Re | Co | 0.791 + 0.00109 A + 0.00223 B - 0.000015 A*A + 0.000014 B*B - 0.000003 A*B |
| Co | Co | Li | 0.904 + 0.00594 A - 0.00517 B - 0.000015 A*A + 0.000014 B*B - 0.000003 A*B |
| Li | Co | Li | 0.882 + 0.00223 A - 0.00523 B - 0.000015 A*A + 0.000014 B*B - 0.000003 A*B |
| Re | Co | Li | 0.943 + 0.00490 A - 0.00480 B - 0.000015 A*A + 0.000014 B*B - 0.000003 A*B |
| Co | Li | Li | -0.015 + 0.00745 A - 0.00043 - 0.000015 A*A + 0.000014 B*B - 0.000003 A*B |
| Li | Li | Li | 0.064 + 0.00374 A - 0.00050 B - 0.000015 A*A + 0.000014 B*B - 0.000003 A*B |
| Re | Li | Li | 0.178 + 0.00641 A - 0.00007 B - 0.000015 A*A + 0.000014 B*B - 0.000003 A*B |
| Co | Re | Li | 0.504 + 0.00544 A + 0.00122 B - 0.000015 A*A + 0.000014 B*B - 0.000003 A*B |
| Li | Re | Li | 0.552 + 0.00173 A + 0.00116 B - 0.000015 A*A + 0.000014 B*B - 0.000003 A*B |
| Re | Re | Li | 0.575 + 0.00440 A + 0.00159 B - 0.000015 A*A + 0.000014 B*B - 0.000003 A*B |
| Co | Co | Re | 0.840 + 0.00319 A - 0.00062 B - 0.000015 A*A + 0.000014 B*B - 0.000003 A*B |
| Li | Co | Re | 0.896 - 0.00052 A - 0.00069 B - 0.000015 A*A + 0.000014 B*B - 0.000003 A*B |
| Re | Co | Re | 0.851 + 0.00216 A - 0.00026 B - 0.000015 A*A + 0.000014 B*B - 0.000003 A*B |

| | | | |
|---|---|---|---|
| Co | Li | Re | 0.261 + 0.00470 A + 0.00411 B - 0.000015 A*A + 0.000014 B*B - 0.000003 A*B |
| Li | Li | Re | 0.417 + 0.00099 A + 0.00404 B - 0.000015 A*A + 0.000014 B*B - 0.000003 A*B |
| Re | Li | Re | 0.425 + 0.00367 A + 0.00448 B - 0.000015 A*A + 0.000014 B*B - 0.000003 A*B |
| Co | Re | Re | 0.469 + 0.00269 A + 0.00577 B - 0.000015 A*A + 0.000014 B*B - 0.000003 A*B |
| Li | Re | Re | 0.595 - 0.00102 A + 0.00570 B - 0.000015 A*A + 0.000014 B*B - 0.000003 A*B |
| Re | Re | Re | 0.5120 + 0.00166 A + 0.00613 B - 0.000015 A*A + 0.000014 B*B - 0.000003 A*B |

Re = Rectilinear, Li = Lines, Co = Concentric

Table S6. Experimental and mathematically predicted elastic modulus.

| **Inner pattern** | **Inner raster (°)** | **Top pattern** | **Bottom pattern** | **Top/ bottom raster** | **Experimental result of UTS (MPa)** | **Predicted result of UTS (MPa)** | **Percentage of error (%)** |
|---|---|---|---|---|---|---|---|
| Re | 90 | Re | Li | 45 | 0.85 | 0.93 | 9.20 |
| Re | 90 | Li | Re | 45 | 0.79 | 0.85 | 6.69 |
| Re | 90 | Re | Re | 90 | 1.18 | 1.18 | 0.00 |
| Re | 45 | Co | Li | 45 | 0.94 | 0.94 | 0.00 |
| Li | 0 | Re | Re | 45 | 0.90 | 0.88 | 1.78 |
| Re | 90 | Co | Re | 45 | 1.03 | 0.92 | 9.98 |
| Li | 45 | Li | Re | 45 | 0.63 | 0.63 | 0.00 |
| Re | 45 | Li | Re | 0 | 0.61 | 0.56 | 8.67 |
| Co | 90 | Re | Re | 45 | 0.90 | 0.86 | 4.54 |
| Re | 0 | Co | Re | 45 | 0.97 | 0.87 | 10.56 |
| Co | 45 | Re | Li | 45 | 0.79 | 0.79 | 0.00 |
| Li | 45 | Re | Re | 90 | 1.12 | 1.13 | 1.43 |
| Re | 45 | Re | Re | 45 | 0.87 | 0.85 | 1.69 |
| Re | 45 | Re | Re | 45 | 0.88 | 0.85 | 2.81 |
| Re | 45 | Re | Re | 45 | 0.85 | 0.85 | 0.16 |
| Re | 45 | Re | Re | 45 | 0.90 | 0.85 | 5.40 |
| Re | 0 | Re | Re | 90 | 1.18 | 1.18 | 0.00 |
| Re | 45 | Re | Re | 45 | 0.83 | 0.85 | 3.31 |
| Co | 45 | Re | Re | 90 | 1.14 | 1.18 | 3.60 |
| Re | 45 | Li | Re | 90 | 1.12 | 1.06 | 4.76 |
| Re | 45 | Re | Co | 90 | 1.14 | 1.11 | 2.42 |
| Re | 0 | Re | Re | 0 | 0.51 | 0.51 | 0.00 |
| Co | 45 | Co | Re | 45 | 0.95 | 0.95 | 0.00 |
| Re | 45 | Co | Re | 0 | 0.81 | 0.92 | 12.58 |
| Co | 45 | Li | Re | 45 | 0.65 | 0.65 | 0.00 |
| Re | 45 | Re | Li | 90 | 1.06 | 0.99 | 7.39 |
| Re | 90 | Re | Re | 0 | 0.54 | 0.54 | 0.00 |
| Re | 45 | Re | Re | 45 | 0.79 | 0.85 | 7.47 |
| Co | 0 | Re | Re | 45 | 0.80 | 0.76 | 5.13 |
| Re | 45 | Re | Co | 0 | 0.84 | 0.81 | 3.30 |
| Li | 45 | Re | Re | 0 | 0.50 | 0.52 | 3.17 |

| | | | | | | | |
|---|---|---|---|---|---|---|---|
| Re | 90 | Re | Co | 45 | 0.85 | 0.88 | 3.24 |
| Re | 45 | Co | Co | 45 | 1.04 | 1.04 | 0.00 |
| Co | 45 | Re | Re | 0 | 0.52 | 0.56 | 7.90 |
| Re | 0 | Re | Co | 45 | 0.89 | 0.92 | 3.09 |
| Li | 45 | Re | Co | 45 | 0.80 | 0.80 | 0.00 |
| Li | 45 | Re | Li | 45 | 0.67 | 0.67 | 0.00 |
| Re | 45 | Li | Co | 45 | 0.73 | 0.73 | 0.00 |
| Co | 45 | Re | Co | 45 | 0.74 | 0.74 | 0.00 |
| Li | 45 | Co | Re | 45 | 0.83 | 0.83 | 0.00 |
| Re | 45 | Re | Li | 0 | 0.82 | 0.74 | 9.58 |
| Re | 0 | Li | Re | 45 | 0.60 | 0.66 | 8.82 |
| Re | 45 | Co | Re | 90 | 0.89 | 0.99 | 11.48 |
| Li | 90 | Re | Re | 45 | 0.67 | 0.65 | 2.39 |
| Re | 0 | Re | Li | 45 | 0.60 | 0.67 | 13.18 |
| Re | 45 | Li | Li | 45 | 0.45 | 0.45 | 0.00 |
| Total error % | | | | | | | 3.82 |

Re = Rectilinear, Li = Lines, Co = Concentric

Table S7. Analysis of variance of yield strength.

| **Source** | **DF** | **Contribution** | **Adj SS** | **Adj MS** | **F-Value** | **P-Value** |
|---|---|---|---|---|---|---|
| Model | 35 | 93.69% | 789.092 | 22.5455 | 4.24 | 0.010 |
| Linear | 8 | 66.16% | 90.014 | 11.2518 | 2.12 | 0.132 |
| A | 1 | 0.11% | 0.335 | 0.3349 | 0.06 | 0.807 |
| B | 1 | 45.34% | 4.889 | 4.8886 | 0.92 | 0.360 |
| C | 2 | 1.90% | 1.763 | 0.8817 | 0.17 | 0.849 |
| D | 2 | 16.39% | 74.657 | 37.3287 | 7.03 | 0.012 |
| E | 2 | 2.42% | 1.163 | 0.5815 | 0.11 | 0.897 |
| Square | 2 | 11.71% | 78.740 | 39.3699 | 7.41 | 0.011 |
| A*A | 1 | 0.27% | 0.438 | 0.4384 | 0.08 | 0.780 |
| B*B | 1 | 11.44% | 68.537 | 68.5373 | 12.90 | 0.005 |
| 2-Way Interaction | 25 | 15.82% | 133.211 | 5.3285 | 1.00 | 0.528 |
| A*B | 1 | 0.00% | 0.019 | 0.0190 | 0.00 | 0.953 |
| A*C | 2 | 1.21% | 8.942 | 4.4708 | 0.84 | 0.459 |
| A*D | 2 | 0.25% | 1.924 | 0.9618 | 0.18 | 0.837 |
| A*E | 2 | 1.35% | 11.404 | 5.7019 | 1.07 | 0.378 |
| B*C | 2 | 1.12% | 0.768 | 0.3838 | 0.07 | 0.931 |
| B*D | 2 | 3.90% | 49.508 | 24.7541 | 4.66 | 0.037 |
| B*E | 2 | 2.86% | 24.081 | 12.0407 | 2.27 | 0.154 |
| C*D | 4 | 0.54% | 5.596 | 1.3989 | 0.26 | 0.895 |
| C*E | 4 | 2.62% | 18.028 | 4.5069 | 0.85 | 0.526 |
| D*E | 4 | 1.97% | 16.558 | 4.1394 | 0.78 | 0.564 |
| Error | 10 | 6.31% | 53.123 | 5.3123 | | |
| Lack-of-Fit | 5 | 5.66% | 47.670 | 9.5339 | 8.74 | 0.016 |
| Pure Error | 5 | 0.65% | 5.453 | 1.0907 | | |
| Total | 45 | 100.00% | | | | |
| $R^2$ = 94.14% | | | | | | |
| $R^2$ - adjusted = 87.72% | | | | | | |

$R^2$ - predicted = 82.16 %

Factors: A=Inner raster, B= T/B raster, C= Inner pattern, D= Top pattern, E= Bottom pattern

Table S8. Regression equation of yield strength with various infill patterns and infill percentage.

| **Inner pattern** | **Top pattern** | **Bottom pattern** | **Regression equation** |
|---|---|---|---|
| Co | Co | Co | 21.78 - 0.0104 A - 0.2085 B + 0.000125 A*A + 0.001561 B*B - 0.000034 A*B |
| Li | Co | Co | 24.52 - 0.0754 A - 0.2029 B + 0.000125 A*A + 0.001561 B*B - 0.000034 A*B |
| Re | Co | Co | 24.59 - 0.0332 A - 0.1925 B + 0.000125 A*A + 0.001561 B*B - 0.000034 A*B |
| Co | Li | Co | 6.27 + 0.0077 A - 0.1015 B + 0.000125 A*A + 0.001561 B*B - 0.000034 A*B |
| Li | Li | Co | 11.48 - 0.0573 A - 0.0960 B + 0.000125 A*A + 0.001561 B*B - 0.000034 A*B |
| Re | Li | Co | 12.19 - 0.0151 A - 0.0856 B + 0.000125 A*A + 0.001561 B*B - 0.000034 A*B |
| Co | Re | Co | 11.00 - 0.0190 A - 0.0744 B + 0.000125 A*A + 0.001561 B*B - 0.000034 A*B |
| Li | Re | Co | 15.84 - 0.0840 A - 0.0688 B + 0.000125 A*A + 0.001561 B*B - 0.000034 A*B |
| Re | Re | Co | 14.84 - 0.0418 A - 0.0584 B + 0.000125 A*A + 0.001561 B*B - 0.000034 A*B |
| Co | Co | Li | 21.57 + 0.0645 A - 0.2206 B + 0.000125 A*A + 0.001561 B*B - 0.000034 A*B |
| Li | Co | Li | 24.23 - 0.0005 A - 0.2150 B + 0.000125 A*A + 0.001561 B*B - 0.000034 A*B |
| Re | Co | Li | 19.71 + 0.0417 A - 0.2046 B + 0.000125 A*A + 0.001561 B*B - 0.000034 A*B |
| Co | Li | Li | 2.52 + 0.0827 A - 0.1136 B + 0.000125 A*A + 0.001561 B*B - 0.000034 A*B |
| Li | Li | Li | 7.66 + 0.0177 A - 0.1081 B + 0.000125 A*A + 0.001561 B*B - 0.000034 A*B |
| Re | Li | Li | 3.78 + 0.0599 A - 0.0977 B + 0.000125 A*A + 0.001561 B*B - 0.000034 A*B |

| | | | |
|---|---|---|---|
| Co | Re | Li | 9.67 + 0.0560 A - 0.0865 B + 0.000125 A*A + 0.001561 B*B - 0.000034 A*B |
| Li | Re | Li | 14.44 - 0.0090 A - 0.0809 B + 0.000125 A*A + 0.001561 B*B - 0.000034 A*B |
| Re | Re | Li | 8.85 + 0.0332 A - 0.0705 B + 0.000125 A*A + 0.001561 B*B - 0.000034 A*B |
| Co | Co | Re | 17.06 + 0.0245 A - 0.1379 B + 0.000125 A*A + 0.001561 B*B - 0.000034 A*B |
| Li | Co | Re | 17.39 - 0.0405 A - 0.1323 B + 0.000125 A*A + 0.001561 B*B - 0.000034 A*B |
| Re | Co | Re | 16.44 + 0.0017 A - 0.1219 B + 0.000125 A*A + 0.001561 B*B - 0.000034 A*B |
| Co | Li | Re | 3.96 + 0.0426 A - 0.0310 B + 0.000125 A*A + 0.001561 B*B - 0.000034 A*B |
| Li | Li | Re | 6.76 - 0.0224 A - 0.0254 B + 0.000125 A*A + 0.001561 B*B - 0.000034 A*B |
| Re | Li | Re | 6.45 + 0.0198 A - 0.0150 B + 0.000125 A*A + 0.001561 B*B - 0.000034 A*B |
| Co | Re | Re | 6.74 + 0.0159 A - 0.0038 B + 0.000125 A*A + 0.001561 B*B - 0.000034 A*B |
| Li | Re | Re | 9.17 - 0.0491 A + 0.0018 B + 0.000125 A*A + 0.001561 B*B - 0.000034 A*B |
| Re | Re | Re | 7.15 - 0.0069 A + 0.0122 B + 0.000125 A*A + 0.001561 B*B - 0.000034 A*B |

Re = Rectilinear, Li = Lines, Co = Concentric

Table S9. Experimental and mathematically predicted yield strength.

| Inner pattern | Inner raster (°) | Top pattern | Bottom pattern | Top/ bottom raster | Experimental result of yield (MPa) | Predicted result of yield (MPa) | Percentage of error (%) |
|---|---|---|---|---|---|---|---|
| Re | 90 | Re | Li | 45 | 11.35 | 12.70 | 11.84 |
| Re | 90 | Li | Re | 45 | 10.66 | 11.59 | 8.77 |
| Re | 90 | Re | Re | 90 | 21.01 | 21.01 | 0.00 |
| Re | 45 | Co | Li | 45 | 15.73 | 15.73 | 0.00 |
| Li | 0 | Re | Re | 45 | 12.07 | 12.41 | 2.83 |
| Re | 90 | Co | Re | 45 | 18.13 | 15.14 | 16.49 |
| Li | 45 | Li | Re | 45 | 7.96 | 7.96 | 0.00 |
| Re | 45 | Li | Re | 0 | 8.53 | 7.60 | 10.96 |
| Co | 90 | Re | Re | 45 | 12.10 | 12.04 | 0.49 |

| | | | | | | | |
|---|---|---|---|---|---|---|---|
| Re | 0 | Co | Re | 45 | 17.10 | 14.12 | 17.47 |
| Co | 45 | Re | Li | 45 | 11.65 | 11.65 | 0.00 |
| Li | 45 | Re | Re | 90 | 20.22 | 19.88 | 1.69 |
| Re | 45 | Re | Re | 45 | 11.16 | 10.74 | 3.78 |
| Re | 45 | Re | Re | 45 | 11.71 | 10.74 | 8.31 |
| Re | 45 | Re | Re | 45 | 11.15 | 10.74 | 3.74 |
| Re | 45 | Re | Re | 45 | 11.56 | 10.74 | 7.12 |
| Re | 0 | Re | Re | 90 | 20.90 | 20.90 | 0.00 |
| Re | 45 | Re | Re | 45 | 9.37 | 10.74 | 14.54 |
| Co | 45 | Re | Re | 90 | 19.82 | 19.88 | 0.30 |
| Re | 45 | Li | Re | 90 | 19.69 | 18.76 | 4.75 |
| Re | 45 | Re | Co | 90 | 20.90 | 20.47 | 2.05 |
| Re | 0 | Re | Re | 0 | 7.15 | 7.15 | 0.00 |
| Co | 45 | Co | Re | 45 | 15.30 | 15.30 | 0.00 |
| Re | 45 | Co | Re | 0 | 13.78 | 16.77 | 21.69 |
| Co | 45 | Li | Re | 45 | 7.83 | 7.83 | 0.00 |
| Re | 45 | Re | Li | 90 | 18.10 | 16.76 | 7.42 |
| Re | 90 | Re | Re | 0 | 7.54 | 7.54 | 0.00 |
| Re | 45 | Re | Re | 45 | 9.46 | 10.74 | 13.45 |
| Co | 0 | Re | Re | 45 | 9.79 | 9.73 | 0.61 |
| Re | 45 | Re | Co | 0 | 13.64 | 13.21 | 3.14 |
| Li | 45 | Re | Re | 0 | 7.55 | 7.21 | 4.52 |
| Re | 90 | Re | Co | 45 | 12.06 | 12.49 | 3.55 |
| Re | 45 | Co | Co | 45 | 17.78 | 17.78 | 0.00 |
| Co | 45 | Re | Re | 0 | 7.65 | 7.71 | 0.78 |
| Re | 0 | Re | Co | 45 | 14.95 | 15.38 | 2.86 |
| Li | 45 | Re | Co | 45 | 12.31 | 12.31 | 0.00 |
| Li | 45 | Re | Li | 45 | 13.73 | 13.73 | 0.00 |
| Re | 45 | Li | Co | 45 | 11.00 | 11.00 | 0.00 |
| Co | 45 | Re | Co | 45 | 10.14 | 10.14 | 0.00 |
| Li | 45 | Co | Re | 45 | 12.96 | 12.96 | 0.00 |
| Re | 45 | Re | Li | 0 | 11.94 | 10.60 | 11.26 |
| Re | 0 | Li | Re | 45 | 8.00 | 8.94 | 11.68 |
| Re | 45 | Co | Re | 90 | 15.31 | 18.30 | 19.52 |
| Li | 90 | Re | Re | 45 | 8.53 | 8.87 | 4.00 |
| Re | 0 | Re | Li | 45 | 7.50 | 8.84 | 17.93 |
| Re | 45 | Li | Li | 45 | 5.43 | 5.43 | 0.00 |
| Total error % | | | | | | | 5.16 |

Re = Rectilinear, Li = Lines, Co = Concentric

Table S10. Analysis of variance of compressive strength.

| **Source** | **DF** | **Contribution** | **Adj SS** | **Adj MS** | **F-Value** | **P-Value** |
|---|---|---|---|---|---|---|
| Model | 35 | 88.77% | 51.7952 | 1.47986 | 2.26 | 0.086 |
| Linear | 8 | 35.09% | 5.8920 | 0.73650 | 1.12 | 0.423 |
| A | 1 | 10.71% | 0.0618 | 0.06182 | 0.09 | 0.765 |
| B | 1 | 0.01% | 0.5714 | 0.57140 | 0.87 | 0.372 |
| C | 2 | 13.07% | 0.9185 | 0.45927 | 0.70 | 0.519 |
| D | 2 | 7.46% | 1.6890 | 0.84450 | 1.29 | 0.318 |

| | | | | | | |
|---|---|---|---|---|---|---|
| E | 2 | 3.83% | 2.5244 | 1.26220 | 1.93 | 0.196 |
| Square | 2 | 1.86% | 0.5721 | 0.28603 | 0.44 | 0.658 |
| A*A | 1 | 0.29% | 0.2179 | 0.21793 | 0.33 | 0.577 |
| B*B | 1 | 1.58% | 0.5442 | 0.54424 | 0.83 | 0.384 |
| 2-Way Interaction | 25 | 51.82% | 30.2342 | 1.20937 | 1.85 | 0.156 |
| A*B | 1 | 0.02% | 0.0092 | 0.00922 | 0.01 | 0.908 |
| A*C | 2 | 7.76% | 3.9790 | 1.98951 | 3.04 | 0.093 |
| A*D | 2 | 7.87% | 0.9892 | 0.49459 | 0.75 | 0.495 |
| A*E | 2 | 6.44% | 3.7586 | 1.87931 | 2.87 | 0.104 |
| B*C | 2 | 7.30% | 4.4774 | 2.23872 | 3.42 | 0.074 |
| B*D | 2 | 1.97% | 1.0737 | 0.53686 | 0.82 | 0.468 |
| B*E | 2 | 0.59% | 0.3462 | 0.17311 | 0.26 | 0.773 |
| C*D | 4 | 0.40% | 0.5284 | 0.13211 | 0.20 | 0.932 |
| C*E | 4 | 2.72% | 1.3097 | 0.32742 | 0.50 | 0.737 |
| D*E | 4 | 16.75% | 9.7712 | 2.44281 | 3.73 | 0.042 |
| Error | 10 | 11.23% | 6.5543 | 0.65543 | | |
| Lack-of-Fit | 5 | 11.08% | 6.4629 | 1.29259 | 70.77 | 0.000 |
| Pure Error | 5 | 0.16% | 0.0913 | 0.01827 | | |
| Total | 45 | 100.00% | | | | |

$R^2$ = 88.77%
$R^2$ - adjusted = 84.46%
$R^2$ - predicted = 80.16 %

Factors: A=Inner raster, B= T/B raster, C= Inner pattern, D= Top pattern, E= Bottom pattern

Table S11. Regression equation of compressive strength with various infill patterns and infill percentage.

| **Inner pattern** | **Top pattern** | **Bottom pattern** | **Regression equation** |
|---|---|---|---|
| Co | Co | Co | 24.72 + 0.0348 A - 0.0311 B - 0.000088 A*A - 0.000139 B*B - 0.000024 A*B |
| Li | Co | Co | 22.70 + 0.0349 A + 0.0084 B - 0.000088 A*A - 0.000139 B*B - 0.000024 A*B |
| Re | Co | Co | 24.32 + 0.0035 A + 0.0068 B - 0.000088 A*A - 0.000139 B*B - 0.000024 A*B |
| Co | Li | Co | 23.86 + 0.0393 A - 0.0153 B - 0.000088 A*A - 0.000139 B*B - 0.000024 A*B |
| Li | Li | Co | 22.03 + 0.0393 A + 0.0241 B - 0.000088 A*A - 0.000139 B*B - 0.000024 A*B |
| Re | Li | Co | 22.70 + 0.0079 A + 0.0225 B - 0.000088 A*A - 0.000139 B*B - 0.000024 A*B |
| Co | Re | Co | 21.77 + 0.0524 A - 0.0113 B - 0.000088 A*A - 0.000139 B*B - 0.000024 A*B |
| Li | Re | Co | 19.69 + 0.0524 A + 0.0281 B - 0.000088 A*A - 0.000139 B*B - 0.000024 A*B |
| Re | Re | Co | 20.81 + 0.0210 A + 0.0265 B - 0.000088 A*A - 0.000139 B*B – 0.000024 A*B |

| | | | |
|---|---|---|---|
| Co | Co | Li | 22.39 + 0.0161 A - 0.0439 B - 0.000088 A*A - 0.000139 B*B - 0.000024 A*B |
| Li | Co | Li | 19.62 + 0.0161 A - 0.0044 B - 0.000088 A*A - 0.000139 B*B – 0.000024 A*B |
| Re | Co | Li | 21.77 - 0.0152 A - 0.0060 B - 0.000088 A*A - 0.000139 B*B - 0.000024 A*B |
| Co | Li | Li | 25.52 + 0.0205 A - 0.0281 B - 0.000088 A*A - 0.000139 B*B - 0.000024 A*B |
| Li | Li | Li | 22.93 + 0.0205 A + 0.0113 B - 0.000088 A*A - 0.000139 B*B - 0.000024 A*B |
| Re | Li | Li | 24.13 - 0.0108 A + 0.0097 B - 0.000088 A*A - 0.000139 B*B - 0.000024 A*B |
| Co | Re | Li | 23.17 + 0.0336 A - 0.0241 B - 0.000088 A*A - 0.000139 B*B - 0.000024 A*B |
| Li | Re | Li | 20.33 + 0.0337 A + 0.0153 B - 0.000088 A*A - 0.000139 B*B - 0.000024 A*B |
| Re | Re | Li | 21.98 + 0.0023 A + 0.0137 B - 0.000088 A*A - 0.000139 B*B - 0.000024 A*B |
| Co | Co | Re | 25.92 - 0.0020 A - 0.0356 B - 0.000088 A*A - 0.000139 B*B - 0.000024 A*B |
| Li | Co | Re | 22.54 - 0.0019 A + 0.0039 B - 0.000088 A*A - 0.000139 B*B - 0.000024 A*B |
| Re | Co | Re | 25.53 - 0.0333 A + 0.0023 B - 0.000088 A*A - 0.000139 B*B - 0.000024 A*B |
| Co | Li | Re | 24.66 + 0.0025 A - 0.0198 B - 0.000088 A*A - 0.000139 B*B - 0.000024 A*B |
| Li | Li | Re | 21.47 + 0.0025 A + 0.0196 B - 0.000088 A*A - 0.000139 B*B - 0.000024 A*B |
| Re | Li | Re | 23.52 - 0.0289 A + 0.0180 B - 0.000088 A*A - 0.000139 B*B - 0.000024 A*B |
| Co | Re | Re | 23.64 + 0.0156 A - 0.0158 B - 0.000088 A*A - 0.000139 B*B - 0.000024 A*B |
| Li | Re | Re | 20.20 + 0.0156 A + 0.0236 B - 0.000088 A*A - 0.000139 B*B - 0.000024 A*B |
| Re | Re | Re | 22.692 - 0.0158 A + 0.0220 B - 0.000088 A*A - 0.000139 B*B - 0.000024 A*B |

Re = Rectilinear, Li = Lines, Co = Concentric

Table S12. Experimental and mathematically predicted compressive strength.

| **Inner pattern** | **Inner raster (°)** | **Top pattern** | **Bottom pattern** | **Top/ bottom raster** | **Experimental result of CS (MPa)** | **Predicted result of CS (MPa)** | **Percentage of error (%)** |
|---|---|---|---|---|---|---|---|
| Re | 90 | Re | Li | 45 | 21.86 | 21.71 | 0.69 |
| Re | 90 | Li | Re | 45 | 21.35 | 20.63 | 3.35 |
| Re | 90 | Re | Re | 90 | 21.22 | 21.22 | 0.00 |
| Re | 45 | Co | Li | 45 | 20.31 | 20.31 | 0.00 |

| | | | | | | | |
|---|---|---|---|---|---|---|---|
| Li | 0 | Re | Re | 45 | 20.69 | 20.98 | 1.41 |
| Re | 90 | Co | Re | 45 | 22.10 | 21.55 | 2.47 |
| Li | 45 | Li | Re | 45 | 21.96 | 21.96 | 0.00 |
| Re | 45 | Li | Re | 0 | 21.32 | 22.04 | 3.35 |
| Co | 90 | Re | Re | 45 | 22.49 | 23.24 | 3.33 |
| Re | 0 | Co | Re | 45 | 25.90 | 25.35 | 2.11 |
| Co | 45 | Re | Li | 45 | 23.09 | 23.09 | 0.00 |
| Li | 45 | Re | Re | 90 | 21.92 | 21.63 | 1.33 |
| Re | 45 | Re | Re | 45 | 22.53 | 22.47 | 0.30 |
| Re | 45 | Re | Re | 45 | 22.24 | 22.47 | 0.99 |
| Re | 45 | Re | Re | 45 | 22.65 | 22.47 | 0.83 |
| Re | 45 | Re | Re | 45 | 22.42 | 22.47 | 0.20 |
| Re | 0 | Re | Re | 90 | 23.55 | 23.55 | 0.00 |
| Re | 45 | Re | Re | 45 | 22.46 | 22.47 | 0.02 |
| Co | 45 | Re | Re | 90 | 22.27 | 21.52 | 3.36 |
| Re | 45 | Li | Re | 90 | 21.72 | 22.43 | 3.29 |
| Re | 45 | Re | Co | 90 | 23.11 | 22.74 | 1.60 |
| Re | 0 | Re | Re | 0 | 22.69 | 22.69 | 0.00 |
| Co | 45 | Co | Re | 45 | 23.72 | 23.72 | 0.00 |
| Re | 45 | Co | Re | 0 | 23.31 | 23.86 | 2.34 |
| Co | 45 | Li | Re | 45 | 23.37 | 23.37 | 0.00 |
| Re | 45 | Re | Li | 90 | 21.77 | 21.92 | 0.69 |
| Re | 90 | Re | Re | 0 | 20.56 | 20.56 | 0.00 |
| Re | 45 | Re | Re | 45 | 22.48 | 22.47 | 0.07 |
| Co | 0 | Re | Re | 45 | 21.90 | 22.65 | 3.42 |
| Re | 45 | Re | Co | 0 | 21.94 | 21.57 | 1.68 |
| Li | 45 | Re | Re | 0 | 21.02 | 20.73 | 1.39 |
| Re | 90 | Re | Co | 45 | 22.43 | 22.80 | 1.65 |
| Re | 45 | Co | Co | 45 | 24.28 | 24.28 | 0.00 |
| Co | 45 | Re | Re | 0 | 24.91 | 24.16 | 3.01 |
| Re | 0 | Re | Co | 45 | 21.35 | 21.72 | 1.73 |
| Li | 45 | Re | Co | 45 | 22.81 | 22.81 | 0.00 |
| Li | 45 | Re | Li | 45 | 22.03 | 22.03 | 0.00 |
| Re | 45 | Li | Co | 45 | 23.56 | 23.56 | 0.00 |
| Co | 45 | Re | Co | 45 | 23.11 | 23.11 | 0.00 |
| Li | 45 | Co | Re | 45 | 22.12 | 22.12 | 0.00 |
| Re | 45 | Re | Li | 0 | 21.75 | 21.90 | 0.69 |
| Re | 0 | Li | Re | 45 | 24.76 | 24.04 | 2.89 |
| Re | 45 | Co | Re | 90 | 22.29 | 22.84 | 2.45 |
| Li | 90 | Re | Re | 45 | 21.29 | 21.58 | 1.37 |
| Re | 0 | Re | Li | 45 | 22.46 | 22.31 | 0.67 |
| Re | 45 | Li | Li | 45 | 23.57 | 23.57 | 0.00 |
| Total error % | | | | | | | 1.15 |

Re = Rectilinear, Li = Lines, Co = Concentric

Table S13. Analysis of variance of compressive modulus.

| **Source** | **DF** | **Contribution** | **Adj SS** | **Adj MS** | **F-Value** | **P-Value** |
|---|---|---|---|---|---|---|
| Model | 35 | 86.81% | 1.34045 | 0.038299 | 1.88 | 0.144 |

| | | | | | | |
|---|---|---|---|---|---|---|
| Linear | 8 | 36.86% | 0.07998 | 0.009998 | 0.49 | 0.837 |
| A | 1 | 24.90% | 0.00922 | 0.009217 | 0.45 | 0.516 |
| B | 1 | 3.26% | 0.02315 | 0.023154 | 1.14 | 0.311 |
| C | 2 | 2.89% | 0.01012 | 0.005061 | 0.25 | 0.785 |
| D | 2 | 2.23% | 0.00857 | 0.004286 | 0.21 | 0.814 |
| E | 2 | 3.56% | 0.00318 | 0.001592 | 0.08 | 0.925 |
| Square | 2 | 1.37% | 0.00597 | 0.002985 | 0.15 | 0.866 |
| A*A | 1 | 0.19% | 0.00134 | 0.001340 | 0.07 | 0.803 |
| B*B | 1 | 1.18% | 0.00595 | 0.005955 | 0.29 | 0.601 |
| 2-Way Interaction | 25 | 48.58% | 0.75023 | 0.030009 | 1.47 | 0.266 |
| A*B | 1 | 0.00% | 0.00000 | 0.000001 | 0.00 | 0.995 |
| A*C | 2 | 5.32% | 0.07199 | 0.035994 | 1.77 | 0.220 |
| A*D | 2 | 4.58% | 0.01827 | 0.009133 | 0.45 | 0.651 |
| A*E | 2 | 2.45% | 0.03788 | 0.018941 | 0.93 | 0.426 |
| B*C | 2 | 18.98% | 0.27403 | 0.137017 | 6.73 | 0.014 |
| B*D | 2 | 0.04% | 0.00052 | 0.000262 | 0.01 | 0.987 |
| B*E | 2 | 0.17% | 0.00263 | 0.001315 | 0.06 | 0.938 |
| C*D | 4 | 7.01% | 0.04518 | 0.011294 | 0.55 | 0.701 |
| C*E | 4 | 2.77% | 0.05969 | 0.014923 | 0.73 | 0.590 |
| D*E | 4 | 7.27% | 0.11231 | 0.028077 | 1.38 | 0.309 |
| Error | 10 | 13.19% | 0.20371 | 0.020371 | | |
| Lack-of-Fit | 5 | 7.20% | 0.11117 | 0.022233 | 1.20 | 0.423 |
| Pure Error | 5 | 5.99% | 0.09254 | 0.018508 | | |
| Total | 45 | 100.00% | | | | |
| $R^2$ = 86.81% | | | | | | |
| $R^2$ - adjusted = 82.14% | | | | | | |
| $R^2$ - predicted = 80.08 % | | | | | | |

Factors: A=Inner raster, B= T/B raster, C= Inner pattern, D= Top pattern, E= Bottom pattern

Table S14. Regression equation of compressive modulus with various infill patterns and infill percentage.

| **Inner pattern** | **Top pattern** | **Bottom pattern** | **Regression equation** |
|---|---|---|---|
| Co | Co | Co | 2.755 + 0.00269 A - 0.00740 B - 0.000007 A*A - 0.000015 B*B - 0.000000 A*B |
| Li | Co | Co | 2.268 - 0.00090 A + 0.00336 B - 0.000007 A*A - 0.000015 B*B - 0.000000 A*B |
| Re | Co | Co | 2.551 - 0.00247 A + 0.00112 B - 0.000007 A*A - 0.000015 B*B - 0.000000 A*B |
| Co | Li | Co | 2.442 + 0.00199 A - 0.00689 B - 0.000007 A*A - 0.000015 B*B - 0.000000 A*B |
| Li | Li | Co | 2.302 - 0.00160 A + 0.00386 B - 0.000007 A*A - 0.000015 B*B - 0.000000 A*B |
| Re | Li | Co | 2.472 - 0.00317 A + 0.00163 B - 0.000007 A*A - 0.000015 B*B - 0.000000 A*B |
| Co | Re | Co | 2.435 + 0.00441 A - 0.00717 B - 0.000007 A*A - 0.000015 B*B - 0.000000 A*B |

| | | | |
|---|---|---|---|
| Li | Re | Co | 2.190 + 0.00082 A + 0.00358 B - 0.000007 A*A - 0.000015 B*B - 0.000000 A*B |
| Re | Re | Co | 2.298 - 0.00076 A + 0.00134 B - 0.000007 A*A - 0.000015 B*B - 0.000000 A*B |
| Co | Co | Li | 2.787 + 0.00251 A - 0.00853 B - 0.000007 A*A - 0.000015 B*B - 0.000000 A*B |
| Li | Co | Li | 2.046 - 0.00108 A + 0.00223 B - 0.000007 A*A - 0.000015 B*B - 0.000000 A*B |
| Re | Co | Li | 2.357 - 0.00265 A - 0.00001 B - 0.000007 A*A - 0.000015 B*B - 0.000000 A*B |
| Co | Li | Li | 2.982 + 0.00180 A - 0.00802 B - 0.000007 A*A - 0.000015 B*B - 0.000000 A*B |
| Li | Li | Li | 2.588 - 0.00178 A + 0.00273 B - 0.000007 A*A - 0.000015 B*B - 0.000000 A*B |
| Re | Li | Li | 2.785 - 0.00336 A + 0.00049 B - 0.000007 A*A - 0.000015 B*B - 0.000000 A*B |
| Co | Re | Li | 2.668 + 0.00422 A - 0.00830 B - 0.000007 A*A - 0.000015 B*B - 0.000000 A*B |
| Li | Re | Li | 2.170 + 0.00063 A + 0.00245 B - 0.000007 A*A - 0.000015 B*B - 0.000000 A*B |
| Re | Re | Li | 2.305 - 0.00094 A + 0.00021 B - 0.000007 A*A - 0.000015 B*B - 0.000000 A*B |
| Co | Co | Re | 2.883 - 0.00045 A - 0.00786 B - 0.000007 A*A - 0.000015 B*B - 0.000000 A*B |
| Li | Co | Re | 2.298 - 0.00404 A + 0.00289 B - 0.000007 A*A - 0.000015 B*B - 0.000000 A*B |
| Re | Co | Re | 2.746 - 0.00562 A + 0.00065 B - 0.000007 A*A - 0.000015 B*B - 0.000000 A*B |
| Co | Li | Re | 2.615 - 0.00116 A - 0.00736 B - 0.000007 A*A - 0.000015 B*B - 0.000000 A*B |
| Li | Li | Re | 2.377 - 0.00475 A + 0.00340 B - 0.000007 A*A - 0.000015 B*B - 0.000000 A*B |
| Re | Li | Re | 2.711 - 0.00632 A + 0.00116 B - 0.000007 A*A - 0.000015 B*B - 0.000000 A*B |
| Co | Re | Re | 2.672 + 0.00126 A - 0.00764 B - 0.000007 A*A - 0.000015 B*B - 0.000000 A*B |
| Li | Re | Re | 2.329 - 0.00233 A + 0.00311 B - 0.000007 A*A - 0.000015 B*B - 0.000000 A*B |
| Re | Re | Re | 2.602 - 0.00390 A + 0.00087 B - 0.000007 A*A - 0.000015 B*B - 0.000000 A*B |

Re = Rectilinear, Li = Lines, Co = Concentric

Table S15. Experimental and mathematically predicted compressive modulus.

| **Inner pattern** | **Inner raster (°)** | **Top pattern** | **Bottom pattern** | **Top/ bottom raster** | **Experimental result of CM (GPa)** | **Predicted result of CM (GPa)** | **Percentage of error (%)** |
|---|---|---|---|---|---|---|---|

| | | | | | | | |
|---|---|---|---|---|---|---|---|
| Re | 90 | Re | Li | 45 | 2.12 | 2.14 | 0.96 |
| Re | 90 | Li | Re | 45 | 2.15 | 2.11 | 2.04 |
| Re | 90 | Re | Re | 90 | 2.15 | 2.15 | 0.00 |
| Re | 45 | Co | Li | 45 | 2.19 | 2.19 | 0.00 |
| Li | 0 | Re | Re | 45 | 2.42 | 2.44 | 0.84 |
| Re | 90 | Co | Re | 45 | 2.31 | 2.18 | 5.43 |
| Li | 45 | Li | Re | 45 | 2.27 | 2.27 | 0.00 |
| Re | 45 | Li | Re | 0 | 2.37 | 2.41 | 1.85 |
| Co | 90 | Re | Re | 45 | 2.27 | 2.36 | 3.87 |
| Re | 0 | Co | Re | 45 | 2.87 | 2.75 | 4.36 |
| Co | 45 | Re | Li | 45 | 2.44 | 2.44 | 0.00 |
| Li | 45 | Re | Re | 90 | 2.39 | 2.37 | 0.85 |
| Re | 45 | Re | Re | 45 | 2.36 | 2.42 | 2.70 |
| Re | 45 | Re | Re | 45 | 2.66 | 2.42 | 8.97 |
| Re | 45 | Re | Re | 45 | 2.48 | 2.42 | 2.34 |
| Re | 45 | Re | Re | 45 | 2.30 | 2.42 | 5.18 |
| Re | 0 | Re | Re | 90 | 2.56 | 2.56 | 0.00 |
| Re | 45 | Re | Re | 45 | 2.30 | 2.42 | 5.13 |
| Co | 45 | Re | Re | 90 | 2.00 | 1.91 | 4.40 |
| Re | 45 | Li | Re | 90 | 2.35 | 2.40 | 1.86 |
| Re | 45 | Re | Co | 90 | 2.29 | 2.25 | 1.76 |
| Re | 0 | Re | Re | 0 | 2.60 | 2.60 | 0.00 |
| Co | 45 | Co | Re | 45 | 2.46 | 2.46 | 0.00 |
| Re | 45 | Co | Re | 0 | 2.35 | 2.48 | 5.32 |
| Co | 45 | Li | Re | 45 | 2.19 | 2.19 | 0.00 |
| Re | 45 | Re | Li | 90 | 2.17 | 2.15 | 0.94 |
| Re | 90 | Re | Re | 0 | 2.19 | 2.19 | 0.00 |
| Re | 45 | Re | Re | 45 | 2.43 | 2.42 | 0.18 |
| Co | 0 | Re | Re | 45 | 2.21 | 2.30 | 3.97 |
| Re | 45 | Re | Co | 0 | 2.29 | 2.25 | 1.77 |
| Li | 45 | Re | Re | 0 | 2.23 | 2.21 | 0.92 |
| Re | 90 | Re | Co | 45 | 2.16 | 2.20 | 1.87 |
| Re | 45 | Co | Co | 45 | 2.45 | 2.45 | 0.00 |
| Co | 45 | Re | Re | 0 | 2.80 | 2.71 | 3.13 |
| Re | 0 | Re | Co | 45 | 2.29 | 2.33 | 1.77 |
| Li | 45 | Re | Co | 45 | 2.34 | 2.34 | 0.00 |
| Li | 45 | Re | Li | 45 | 2.27 | 2.27 | 0.00 |
| Re | 45 | Li | Co | 45 | 2.36 | 2.36 | 0.00 |
| Co | 45 | Re | Co | 45 | 2.27 | 2.27 | 0.00 |
| Li | 45 | Co | Re | 45 | 2.20 | 2.20 | 0.00 |
| Re | 45 | Re | Li | 0 | 2.27 | 2.25 | 0.90 |
| Re | 0 | Li | Re | 45 | 2.78 | 2.73 | 1.58 |
| Re | 45 | Co | Re | 90 | 2.29 | 2.42 | 5.46 |
| Li | 90 | Re | Re | 45 | 2.15 | 2.17 | 0.95 |
| Re | 0 | Re | Li | 45 | 2.27 | 2.29 | 0.90 |
| Re | 45 | Li | Li | 45 | 2.61 | 2.61 | 0.00 |
| Total error % | | | | | | | 1.79 |

Re = Rectilinear, Li = Lines, Co = Concentric
